# Chiral (Stereoselective) Drugs, Asymmetric Synthesis, and Racemic Resolution Methods

**DOI:** 10.1002/chir.70109

**Published:** 2026-06-17

**Authors:** Burcu Karayavuz, Cigdem Karaaslan Kirmizioglu

**Affiliations:** ^1^ Faculty of Gulhane Pharmacy, Department of Pharmaceutical Chemistry University of Health Sciences Ankara Turkey

## Abstract

Chirality is of great importance in drug development since both biological targets in the organism and the majority of pharmacologically active compounds created today are chiral. The synthesis and resolution of chiral compounds are critical steps while developing chiral drugs, as the chirality of a molecule can significantly affect its efficacy, safety (side effects and toxicity), and metabolism. While one enantiomer has therapeutic properties, the other enantiomer might be harmful, toxic, or ineffective. In recent years, the trends towards developing single enantiomer drugs have increased in the pharmaceutical industry. Various synthetic strategies and racemic resolution techniques are employed to obtain optically pure chiral drugs. Regulatory authorities also give more priority to assessing the biological efficacy of each chiral drug's enantiomer. This review summarizes the asymmetric synthesis and racemic resolution methods for producing pure enantiomeric pharmaceutical active ingredients and their synthetic intermediates, with examples from the pharmaceutical industry. By examining current approaches and challenges in the field, we aim to provide a comprehensive understanding of the processes that ensure the production of safe and effective chiral‐specific drugs.

## Graphical abstract

Chirality is crucial in drug development since both biological targets in the organism and the majority of pharmaceutical compounds are chiral. The synthesis and resolution of chiral compounds are critical steps while developing chiral drugs. Asymmetric synthesis and racemic resolution are the two most common methods for obtaining enantiopure drugs.

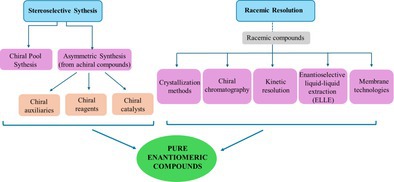

## 1 Introduction

**FIGURE 1:**
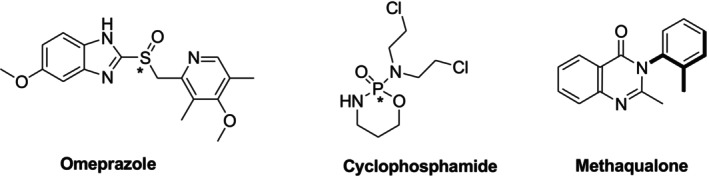
Representative examples of noncarbon stereogenic elements and axial chirality: sulfur‐centered chirality in omeprazole, phosphorus‐centered chirality in cyclophosphamide, and chirality arising from hindered rotation in Methaqualone.

Molecular chirality was first demonstrated by the French scientist Louis Pasteur in 1848 by mechanically separating the two isomers of sodium ammonium tartrate and examining it in a polarimeter [1]. A key structural characteristic of many biological targets and pharmacologically active substances is chirality, which is the inability to superimpose an object's mirror image. The carbon atom forms an asymmetric center (stereogenic center) when it has four distinct substituents. The ability to function as an asymmetric center is not limited to carbon atoms. Sulfur, nitrogen, and phosphorus atoms can also lead to the formation of chiral compounds under certain circumstances. Examples include the sulfur in omeprazole, the phosphorus in cyclophosphamide, and the chirality due to hindered rotation in methaqualone (Figure 1).

The body, which contains a large number of homochiral biological targets such as proteins, enzymes, amino acids, carbohydrates, nucleic acids, is stereochemistry sensitive. Basic physiological functions such as the transport of enantiomers across membranes, their metabolism, and their interaction with receptors can occur in a stereoselective manner. Sometimes, the administration of a single stereoisomer can result in the formation of multiple stereoisomers in metabolic processes within the body. For example, in ibuprofen, approximately 60% of the *R*‐enantiomer has been shown to be converted to the pharmacologically more active *S*‐enantiomer via metabolic chiral inversion [2]. As another example, thalidomide undergoes rapid racemization under physiological conditions, with continuous stereochemical interconversion between its enantiomers [3]. Therefore, it is very important to consider the chirality when developing drugs. While one isomer has therapeutic benefits, the other might be less active or inactive, and even produce undesirable or toxic effects [4, 5]. A well‐known illustration that highlights the significance of chirality in medication development is the thalidomide disaster. Originally developed as a sedative in the late 1950s, thalidomide has also been used for the treatment of nausea during pregnancy. Thalidomide, which previously existed as a racemic mixture, was later discovered to only be therapeutic in the *R*‐isomer form, with the *S*‐isomer causing teratogenic (mutagenic) effects, which caused thousands of children in Europe to be born with birth abnormalities (phocomelia). Due to teratogenicity and neuropathy, the use of this drug was abandoned in 1961 [6].

**FIGURE 2:**
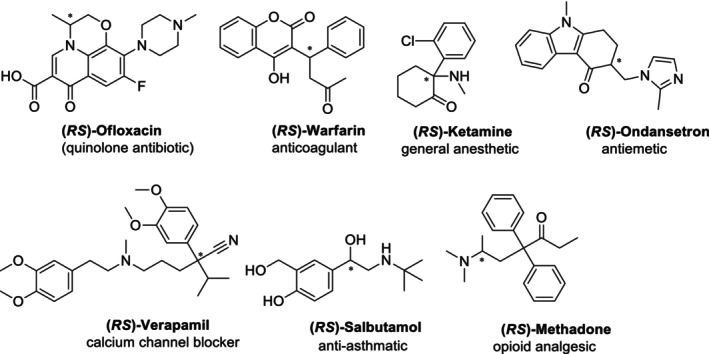
Examples of chiral drugs from different therapeutic classes.

The pharmaceutical industry is becoming more and more interested in chiral medicines. Approximately 50%–65% of currently used drugs consist of chiral molecules, and most of them are still marketed in the racemate form. Examples of drugs belonging to different therapeutic classes and marketed in racemate form are given in Figure 2 [4, 7]. Most racemic medications contain one main bioactive enantiomer, known as the eutomer; the other is hazardous, inactive, or less active (distomer), or it may have other pharmacological effects that are either wanted or unwanted. The enantiomers of a chiral drug may display distinct pharmacokinetic and pharmacological properties because of the chiral nature of the body (chiral proteins, amino acids, enzymes, etc.); therefore, employing medications with a single enantiomer rather than a racemate may improve the treatment's effectiveness and/or safety [8].

**FIGURE 3:**
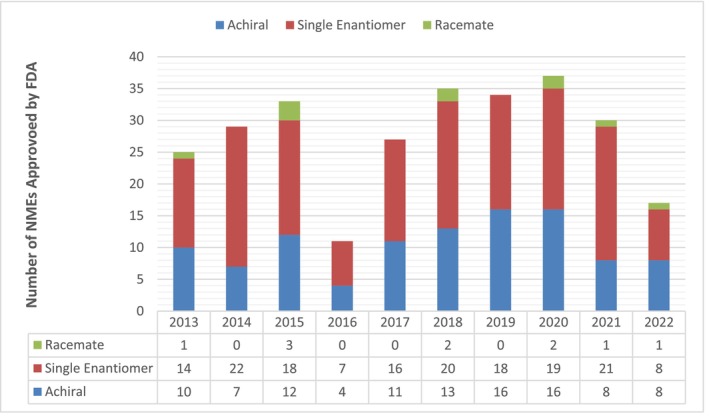
Comparative analysis of the number of FDA‐approved medications for pure enantiomers, racemates, and achiral pharmaceuticals from 2013 to 2022. Data were compiled from McVicker and O'Boyle [8] and edited by the author using FDA approval databases.

**FIGURE 4:**
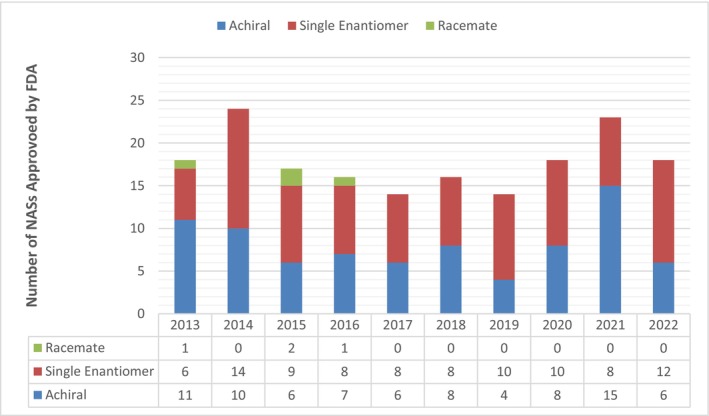
Comparative analysis of the number of EMA‐approved medications for pure enantiomers, racemates, and achiral pharmaceuticals from 2013 to 2022. Data were compiled from McVicker and O'Boyle [8] and edited by the author using EMA approval databases.

Along with the technological advances in chiral resolution and asymmetric synthesis methods, the US Food and Drug Administration (FDA) has demanded the evaluation of biological activity of each enantiomer in racemic medications before they are placed on the market since 1992, and supports the development of new pure enantiomeric drugs [9]. In addition, now FDA is increasingly approving pure enantiomeric drugs over racemates. In 2024, McVicker and O'Boyle provided an analysis that categorized newly approved FDA small molecule drugs according to their chirality [10]. According to this analysis, between 2013 and 2022, FDA approved only 10 drugs in racemic form. It was reported that racemic drug approvals decreased from 11% to 3.6% compared to the previous decade (2003–2012), while the approval rate for single‐enantiomer drugs increased from 57% to 59% during the same period. The European Medicines Agency (EMA) has similar reduced racemic drug approvals and has shown an increasing trend towards single‐enantiomer drugs. Only four racemic new active substances (NAS) received EMA clearance between 2013 and 2022, and the agency has not authorized a new racemic medication since Lesinurad's 2016 approval. EMA approved 41 achiral and 48 single‐enantiomer drugs between 2018 and 2022. An analysis of the quantity of racemic, achiral, and single enantiomer medications that were authorized by the FDA and EMA between 2013 and 2022 is given in Figures 3 and 4, respectively.

**FIGURE 5:**
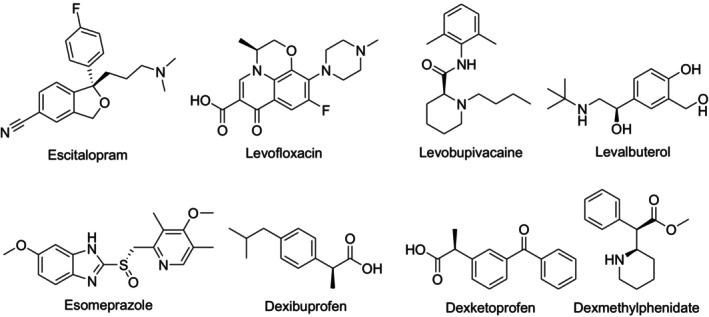
Examples of clinically used chiral switches.

The term chiral switch refers to the reintroduction of a chiral drug currently marketed as a racemate to a single enantiomeric form. Chiral switching has been used by pharmaceutical companies since the 2000s as one of their marketing strategies for chiral drugs [11]. This approach may provide potential benefits such as reduced side effects, increased selectivity for the receptor, reduced dose of administered drug, improved pharmacokinetic profile, reduced drug–drug interactions, and decreased interindividual variability of therapeutic response. This strategy is also used by pharmaceutical companies to extend patents and protect against generic competition. There are clinically successful examples of chiral switching [12, 13, 14] (Figure 5). However, comparison of a single enantiomeric drug with its racemic equivalent is not required to get FDA and EMA approval. The superior efficacy of a single enantiomeric drug over placebo is sufficient to enter the market. Therefore, there is no definite evidence that positive changes will always occur in treatment by chiral switch [8, 15].

**FIGURE 6:**
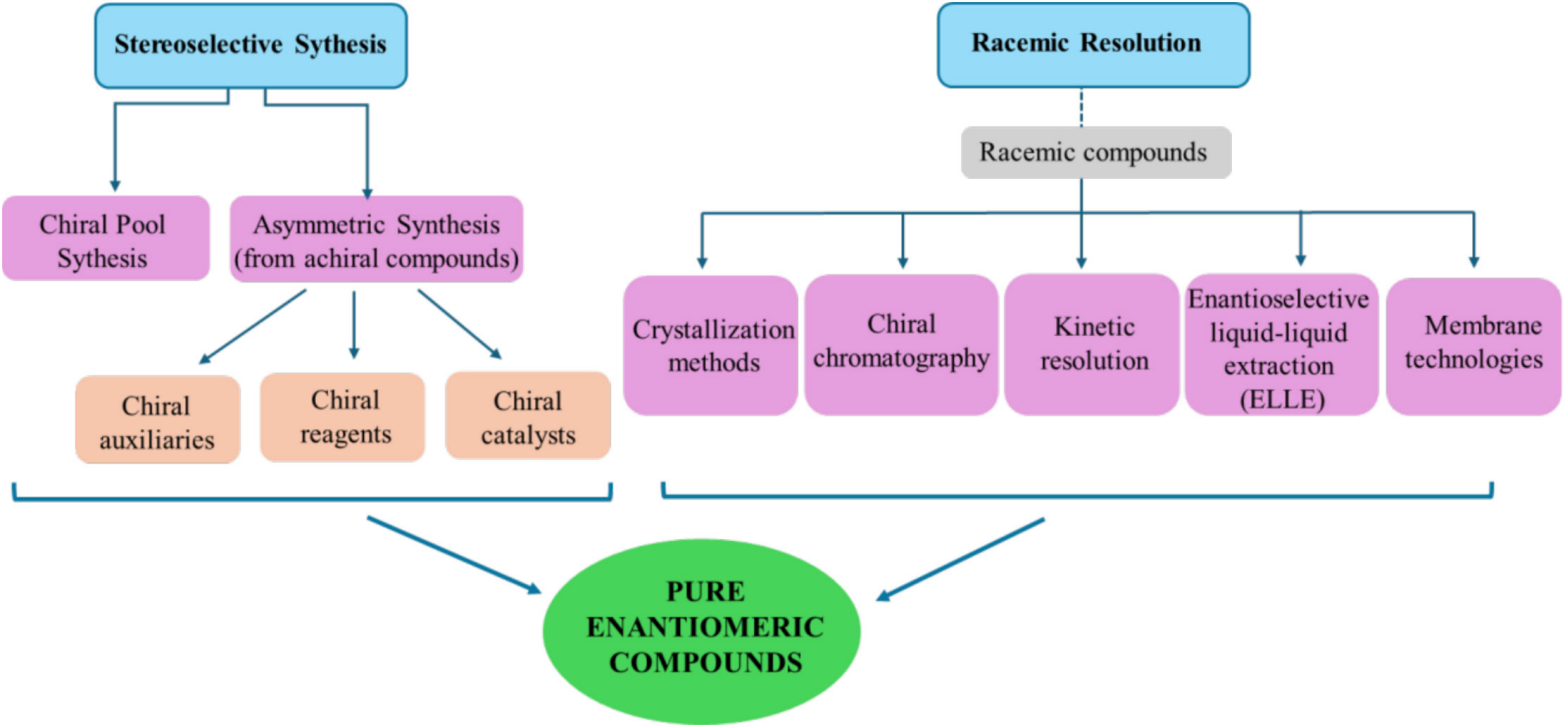
Methods for obtaining pure enantiomeric compounds.

In order to assess the individual biological activities of enantiomers of chiral drugs, which show particularly promising therapeutic efficacy, it is necessary to obtain each enantiomer in pure form. There are basically two approaches to achieve pure enantiomer chiral pharmaceuticals (Figure 6):

- Stereoselective synthesis from chiral compounds of natural origin (chiral pool synthesis) or asymmetric synthesis from achiral compounds,
- Separation of enantiomers from racemic mixtures (racemic resolution) [16].

Chiral pool synthesis (chiron approach) refers to a synthetic process that uses natural enantiopure substances (e.g., carbohydrates, terpenes, amino acids, alkaloids, and hydroxy acids) from the origin of plants or animals as a starting compound for the synthesis of a target molecule. The other method for stereoselective production of enantiomers is asymmetric reactions of achiral compounds with chiral agents (a chiral auxiliary, reagent, or catalyst) (asymmetric synthesis). Another way to get pure enantiomers is racemic resolution, which means separation of racemates into their pure enantiomers. Having the same physicochemical properties (boiling point, melting point, solubility, etc.) prevents the enantiomers from being easily separated by conventional separation methods such as distillation, extraction, and chromatography. Therefore, racemic resolution is accomplished using special methods such as crystallization via diastereomeric salt formation, chiral chromatography, kinetic resolution, enantioselective extraction, and membrane‐based technologies. Small quantities of many compounds are required for bioscreening in the early phases of drug development. At this point, stereoselective synthesis might not be cost‐ and time‐effective. Racemic resolution has the advantage of enabling preparatory‐scale separation of enantiomers required for biological testing [17, 18].

In recent years, several comprehensive reviews have addressed chiral drugs, enantioselective synthesis, and racemic resolution strategies. While these studies have significantly contributed to the field, most of them primarily focus on either fundamental principles or specific methodological aspects. The present review intends to provide a comprehensive and updated perspective by integrating recent developments across synthetic strategies, resolution techniques, and their pharmaceutical applications. In particular, this review incorporates recent regulatory trends in FDA and EMA approvals, highlights the increasing dominance of single‐enantiomer drugs, and includes emerging approaches (2020–2025) such as continuous flow synthesis, machine learning (ML)‐assisted catalyst design, and electrochemical asymmetric synthesis. Furthermore, it emphasizes the translation of chiral technologies from laboratory research to industrial‐scale production, supported by up‐to‐date examples from the pharmaceutical industry. By combining methodological advances with practical and regulatory perspectives, this work seeks to complement existing literature and provide a more comprehensive and current overview of chirality in modern drug development.

## 2 Synthesis of Pure Enantiomeric Compounds

Synthesis of optically active compounds can be carried out using natural enantiopure compounds or from prochiral compounds (an achiral compound that can change to a chiral state in a single step) in the existence of a chiral auxiliary, a chiral reagent, or a chiral catalyst. Chiral pool and asymmetric synthesis approaches have been used to obtain many pharmaceutical compounds [19, 20, 21]. In this section, basic principles of chiral pool synthesis and asymmetric synthesis approaches and examples of their applications in drug development are presented.

### 2.1 Chiral Pool Synthesis

The chiral pool method employs naturally existing chiral compounds as starting materials for the production of more intricate compounds, providing an optically active raw material. They are useful for industrial uses as they are commercially available low‐cost starting materials. Carbohydrates (D‐glucose, D‐sucrose, etc.), amino acids (L‐proline, L‐serine, L‐phenylalanine, etc.), terpenes (*α*‐pinene, (−)‐l‐menthol, etc.), α‐hydroxy acids (L‐lactic acid), and alkaloids (quinidine, quinine, etc.) are some of the significant components of the chiral pool [22, 23, 24]. Applications of the chiral pool for the production of drugs and drug intermediates have been reported in the literature [25, 26, 27, 28].

**FIGURE 7:**
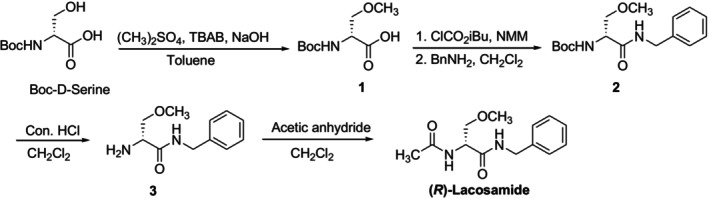
Synthetic pathway of lacosamide developed by UCB Pharma.

Vimpat, an antiepileptic drug manufactured by UCB Pharma, contains lacosamide, also known chemically as (*R*)‐2‐acetamido‐*N*‐benzyl‐3‐methoxypropionamide [29, 30]. Several synthetic routes have been reported for the stereoselective production of (*R*)‐lacosamide. [31, 32]. The synthetic method described by UCB Pharma, which involved the *O*‐methylation of an *N*‐Boc‐d‐serine, benzylamide production, *N*‐deprotection, and *N*‐acetylation, served as the foundation for its commercial routes (Figure 7) [33].

**FIGURE 8:**
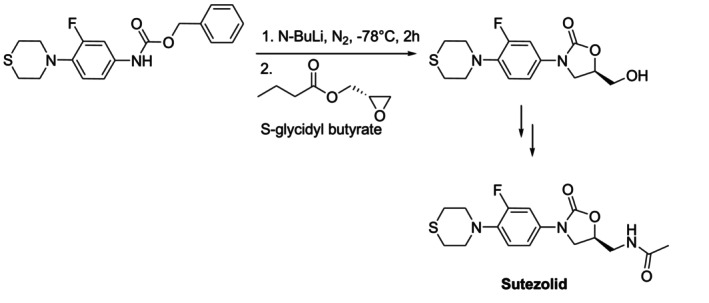
Chiral pool synthesis of sutezolid.

Amino acids have been widely utilized as a chiral pool enabling the production of heterocycles because they are readily available in enantiomerically enriched form and have a range of chemically convertible functional groups [34]. It has been reported that sutezolid, a novel oxazolidinone derivative currently in clinical trials (Phase IV) against extremely drug‐resistant tuberculosis [35, 36], was synthesized from the chiral pool (*S*‐glycidyl butyrate) (Figure 8) [37].

**FIGURE 9:**
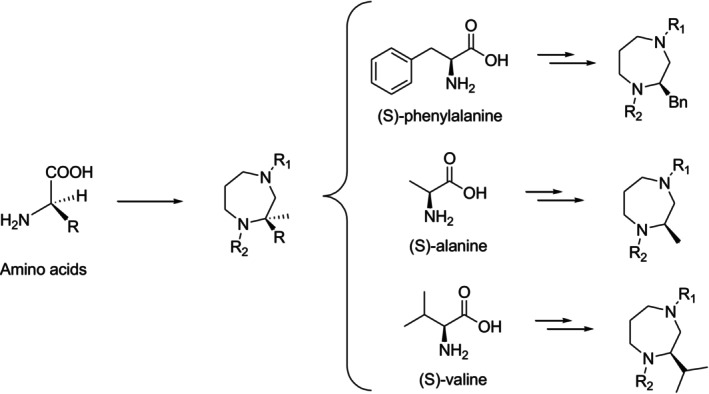
An amino acid–based chiral pool synthesis of 1,2,4‐trisubstituted 1,4‐diazepanes.

In another instance, Fanter et al. produced 1,4‐diazepanes as ligands for the σ1 receptor by beginning with enantiopure amino acids and adding different substituents to the 1, 2, and 4‐positions (Figure 9) [38].

**FIGURE 10:**
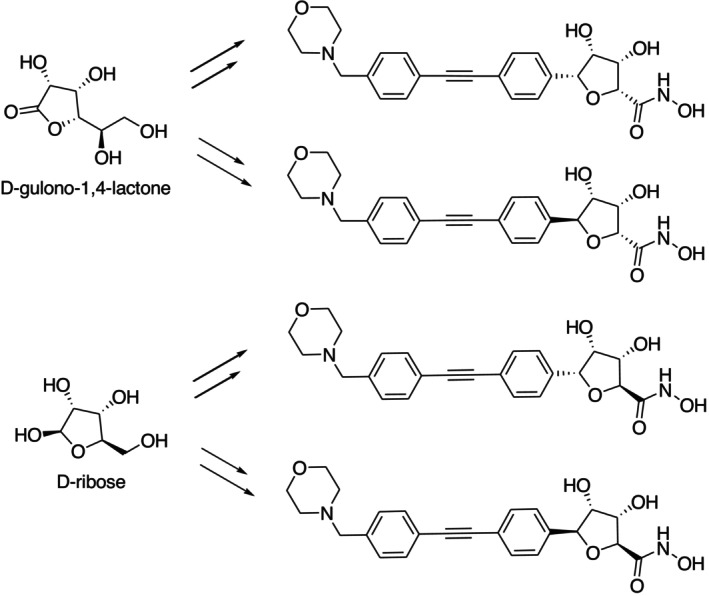
Bacterial deacetylase LpxC inhibitors synthesized from the chiral pool of D‐gulono‐1,4‐lactone and D‐ribose.

Dreger et al. reported synthesizing four (3*S*,4*R*)‐structured dihydroxytetrahydrofuran derivative chemicals using chiral pools, beginning with D‐gulono‐1,4‐lactone and D‐ribose. (Figure 10) [39].

**FIGURE 11:**
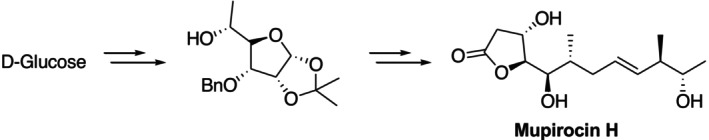
Synthesis of mupirocin from D‐glucose.

D‐glucose was utilized as a chiral pool material in the complete synthesis of mupirocin H described by Udawant et al. Mupirocin H is one of the best topical antibiotics for eliminating nasal *Staphylococcus aureus* , even methicillin‐resistant *S. aureus* , in addition to being highly efficient in treating skin infections (Figure 11) [40].

**FIGURE 12:**
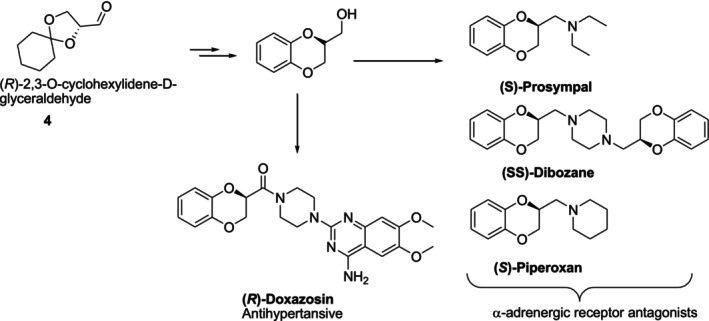
Chiral pool approach for 1,4‐benzodioxane‐containing bioactive compound synthesis.

Cyclohexylideneglyceraldehyde is a useful chiral synthon for the production of chiral chemicals and is typically produced from D‐mannitol. Starting from the inexpensive and widely available chiral compound cyclohexylideneglyceraldehyde **4**, 1,4‐benzodioxane, a precursor to bioactive molecules, such as the antihypertensive agent doxazosin and the α‐blockers piperoxane, prosympal, and dibosan, has been obtained (Figure 12) [41].

The chiral pool strategy could be used for the creating of complex bioactive compounds. The relatively cheap and high optical purity of natural compounds are the advantageous aspects of the method. However, the enantiopure precursors required for the desired stereoisomer are not always available in the chiral pool. In the absence of natural sources, racemic resolution, use of a chiral auxiliary, or asymmetric catalysis may be employed to create stereocenters.

### 2.2 Asymmetric Synthesis

Asymmetric synthesis or stereoselective synthesis refers to processes that result in the formation of one or more novel stereocenter configurations. The generation of the stereogenic center is induced using a chiral auxiliary, catalyst, or reagents. Asymmetric synthesis involves the enantioselective conversion of an achiral molecule into a chiral molecule or the diastereoselective conversion of a chiral molecule into a new chiral molecule [42]. To create an enantiopure medicinal compound, asymmetric synthesis has been extensively used. The following is a description of various asymmetric synthesis techniques for producing enantiopure medicinal compounds.

#### 2.2.1 Chiral Auxiliary

Many chiral auxiliaries are fundamental building blocks for the synthesis of extremely complex structures and are sourced from substances that exist naturally like terpenes, amino acids, and carbohydrates. Chiral auxiliaries are molecules that can attach temporarily to the beginning compound and cause chirality in a synthetic process. Binding of the chiral auxiliary to the achiral substrate allows the next step of the reaction to proceed with considerable stereoselectivity. After the reaction, the chiral auxiliary is eliminated or recycled [43]. For each reaction, there are various chiral auxiliary options that can be selected. Auxiliaries should produce high chemical yields and great enantioselectivity, and their addition and removal operations should be simple to carry out or take place under temperate conditions. Furthermore, it is important that the chosen chiral auxiliary does not affect the newly created stereogenic centers or those already present in the precursor compound. In addition, the recoverability and re‐employment of the chiral auxiliary are crucial and should be taken into consideration when designing the synthetic pathway because they are frequently expensive and employed in stoichiometric quantities [44].

**FIGURE 13:**
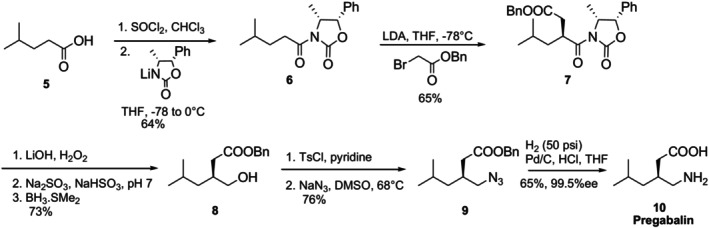
Pfizer's asymmetric synthesis of pregabalin.

Asymmetric synthesis via chiral auxiliary has been effectively employed in the synthesis of many drug molecules used in clinical practice. In asymmetric total synthesis, Evans' chiral auxiliaries (commercially available chiral oxazolidinones) are among the most frequently employed auxiliaries. Evans' chiral auxiliaries have been used in numerous reactions, including α‐alkylation, 1,4‐addition, Michael additions, intramolecular Diels‐Alder cycloadditions, and additions to C‐O and C‐N bonds [43, 44, 45, 46]. Evans' chiral auxiliary was employed in the first enantioselective synthesis of the anticonvulsant drug pregabalin **10**, which Pfizer developed to treat neuropathic pain (Figure 13) [47, 48]. By using this method, 4‐methylpentanoic acid **5** was transformed into the corresponding acid chloride and then Evans' chiral auxiliary was connected to it. Acyloxazolidinone **6** was alkylated with bromoacetate to create the highly enantioselective benzyl ester **7**. This chiral auxiliary was removed from **7** by basic hydrolysis, and the resultant carboxylic acid was reduced with BH3SMe2 to produce the matching alcohol **8**. The compound azide **9** was produced by a tosylation process and sodium azide treatment of the tosylate that was produced. Eventually, reduction in a hydrogen atmosphere and Pd/C catalysis produced pregabalin **10** in 65% yield and with 99.5% enantioselectivity.

**FIGURE 14:**
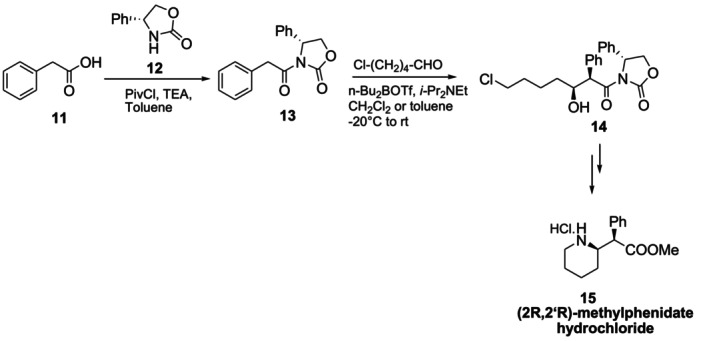
Chiral auxiliary mediated synthesis of (2*R*,2′*R*)‐methylphenidate hydrochloride.

Novartis chemists Prashad et al. reported the nine‐step chiral auxiliary‐controlled asymmetric synthesis of (2*R*,2′*R*)‐(+)‐*threo*‐methylphenidate hydrochloride **15** (Ritalin). In the method, phenylacetic acid **11** was first linked with the chiral auxiliary (*R*)‐4‐phenyl‐*N*‐phenylacetyl‐2‐oxazolidinone **12** in an environment of pivaloyl chloride and triethylamine. The desired single diastereomer **14** was obtained as a result of the aldol condensation reaction of the obtained molecule **13** with 5‐chloropentanal (Figure 14) [49].

**FIGURE 15:**
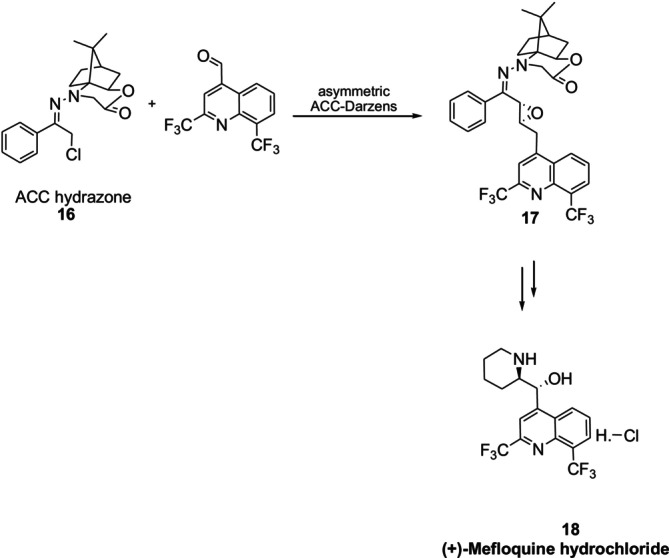
Asymmetric synthesis of (+)‐mefloquine hydrochloride using chiral *n*‐amino cyclic carbamate hydrazones.

Knight et al. reported a method for total asymmetric synthesis of the (+)‐enantiomer of the antimalarial drug Meflokin **18**. The asymmetric conversion occurred by the asymmetric Darzens reaction of a chiral α‐chloro‐*N*‐amino cyclic carbamate hydrazone **16** (Figure 15) [50].

**FIGURE 16:**
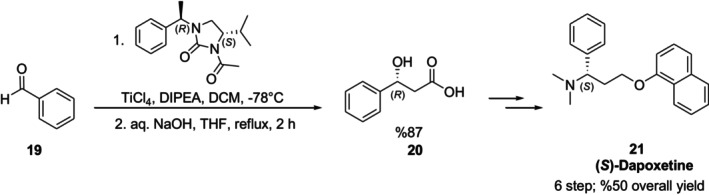
Enantioselective synthesis of (*S*)‐dapoxetine using imidazolidin‐2‐one chiral auxiliary.

Dapoxetine **21** is a selective serotonin reuptake inhibitor used to treat premature ejaculation [51]. Dapoxetine's (*S*) enantiomer has a 3.5‐fold higher potency than the opposite [52]. Khatik et al. created a technique for the enantioselective synthesis of (*S*)‐dapoxetine using an imidazolidin‐2‐one chiral auxiliary mediated acetate aldol reaction. In the method, the chiral auxiliary was first treated with titanium tetrachloride and *N,N*‐diisopropylethylamine (DIPEA) at −78°C. The following reaction with benzaldehyde **19** produced the syn:anti acetate aldol product with a diastereoselectivity of 99:01, respectively. With the use of aqueous NaOH, the auxiliary was cleaved from the *syn* aldol adduct to produce (*R*)‐3‐hydroxy‐3‐phenylpropanoic acid **20**, the key chiral intermediate of dapoxetine synthesis (Figure 16) [53].

**FIGURE 17:**
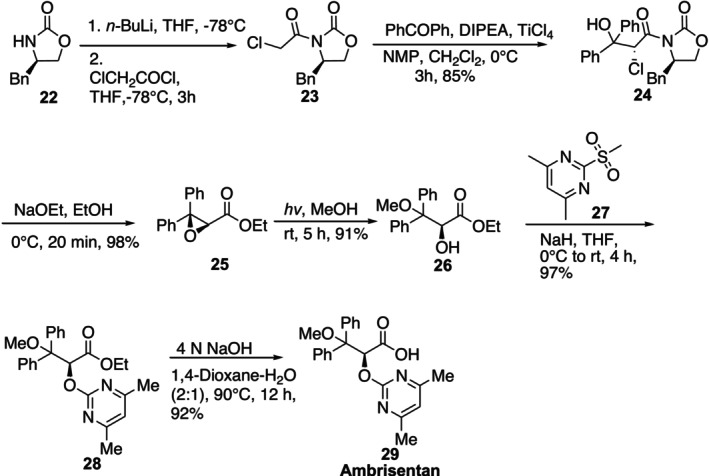
Enantioselective synthesis of Ambrisentan.

Ambrisentan is an oral drug that is a very specific antagonist of the endothelin‐1 type A receptor and is indicated to treat pulmonary arterial hypertension (PAH) [54]. Ambrisentan **29** was synthesized in a very efficient and enantioselective method by Madhu et al. (Figure 17) [55]. The essential processes include chiral auxiliary mediated enantioselective epoxidation (Azerad procedure), photochemical regioselective epoxide opening, and base‐mediated ester hydrolysis. In the total synthesis of ambresentan, (*R*)‐4‐benzyloxazolidin‐2‐one **22** is transformed into the derivative of chloro‐acetyl **23** with the use of chloroacetyl chloride and n‐BuLi in anhydrous tetrahydrofuran at −78°C. When benzophenone and chiral auxiliary **23** were coupled under Crimmins modified Evans aldol conditions, a 20:1 ratio of diastereomeric mixture was produced. Upon recrystallization, the pure isomer **24** was obtained in 85% yield as a colorless solid. A modified Azerad process using NaOEt in EtOH and in situ trans esterification and ring closure was used to obtain *trans* epoxy ester **25**. Using UV light with a wavelength of 253.7 nm, the compound 4 was photoirradiated in methanol to produce the ‐hydroxyester **26** with a 91% yield. The resultant ‐hydroxy ester **26** was processed with 4,6‐dimethyl‐2‐(methylsulfonyl)pyrimidine **27** and NaH in THF to produce ester **28** in 97% yield. Ultimately, the target molecule **29** was produced with a 92% yield using a basic hydrolysis of the ester functionality.

**FIGURE 18:**
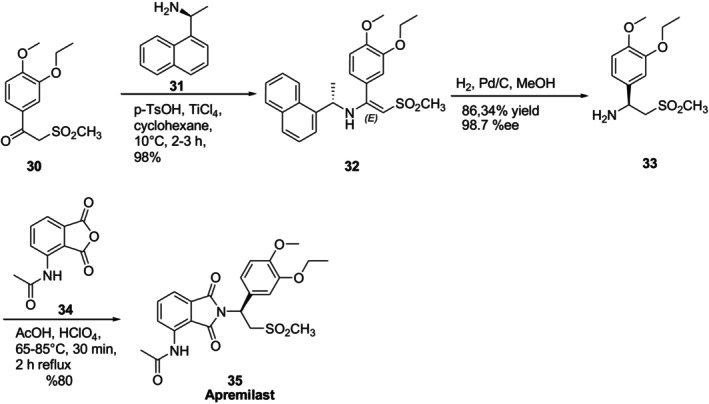
Synthesis of Apremilast using chiral auxiliary (*R*)‐(+)‐1‐(1‐naphthyl)ethylamine.

Apremilast (Otezla) is an anti‐inflammatory drug that is a small molecule phosphodiesterase 4 (PDE4) inhibitor. It is indicated for the management of psoriatic arthritis and psoriasis [56, 57]. Xinfa Pharmaceutical scientists reported a synthetic route for Apremilast, also known as *N*‐[2‐[(1S)‐1‐(3‐ethoxy‐4‐methoxyphenyl)‐2‐methylsulfonylethyl]‐1,3‐dioxoisoindol‐4‐yl]acetamide **35** (Figure 18) [58, 59]. The chiral auxiliary in this route was (*R*)‐(+)‐1‐(1‐naphthyl)ethylamine **31**. Under acid catalyst, compound **30** interacted with chiral auxiliary **31** to produce (*S,E*)‐1‐(3‐ethoxy‐4‐methoxyphenyl)‐2‐(methylsulfonyl)‐*N*‐(1‐(naphthalen‐1‐yl)ethyl) ethan‐1‐amine **32** in 98% yield. Catalytic hydrogenation of compound **32** gave apremilast chiral key intermediate **33**. Apremilast **35** was produced by condensation of **33** and **34** in an acidic environment.

**FIGURE 19:**
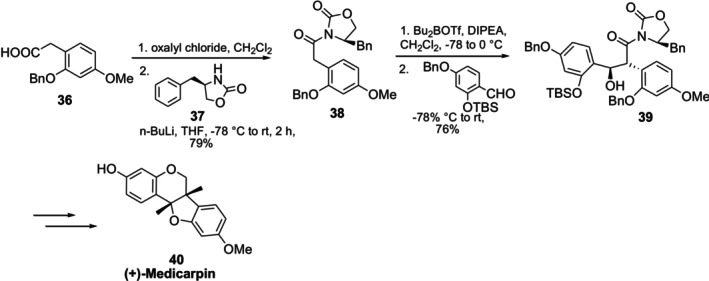
Synthesis of (+)‐Medicarpin using chiral auxiliary (*R*)‐4‐benzyl‐2‐oxazolidinone.

Medicarpin is a pterocarpan, which is an isoflavonoid derivative, and is composed of 9‐methoxy‐6a,11a‐dihydro‐6*H*‐benzo[4,5]furo‐[3,2‐c]chromen‐3‐ol. Medicarpin has been studied as a possible therapy for postmenopausal osteoporosis, mostly in a racemate form [60, 61]. Moreover, medicarpin could increase the sensitivity of multi‐drug resistant P388 leukemia cancer cells to chemotherapy and has been linked to possible anticancer effects [62]. In 2017, Yang et al. demonstrated the asymmetric synthesis of (+)‐Medicarpin **40** with an overall yield of 11%. Through condensation with a chiral oxazolidinone auxiliary **37**, the two stereogenic centers were produced in a single step [63] (Figure 19).

**FIGURE 20:**
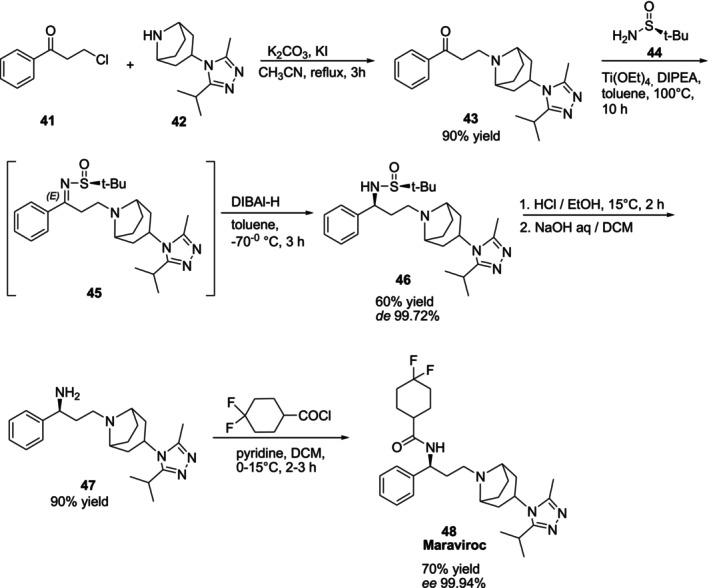
Synthesis of Maraviroc utilizing chiral auxiliary (*S*)‐*tert*‐butanesulfinamide **44**.

Maraviroc **48** is a chemokine receptor type 5 (CCR5) inhibitor that is utilized to treat HIV‐1 infection. In 2019, Zhu et al. developed the asymmetric synthesis of the anti‐HIV drug Maraviroc by employing (*S*)‐*tert*‐butanesulfinamide **44** as chiral auxiliary. It is a desirable chiral auxiliary in the synthesis of chiral amine compounds because of its great diastereoselectivity and easy removal of the *N*‐*tert*‐butanesulfinyl group. The synthetic route is given in Figure 20 [64].

Chiral auxiliaries enable highly stereocontrolled reactions, but these reactions require an excessive number of them and more processes to remove them after the reaction, which can make costs and overall yield lower. Therefore, this approach is generally less preferred in the early stages of new drug discovery processes that require rapid derivatization and aim for high yield in structure–activity relationship studies.

#### 2.2.2 Chiral Reagent

Asymmetric synthesis using chiral reagents is still important as a technique, but it is not frequently employed because the chiral reagents are quite expensive when used in stoichiometric amounts. An asymmetric center is added while using the chiral reagent to replace the compound's original center. In contrast to the chiral auxiliary, it does not require extra binding and removal procedures. In general, this technique relies on the transfer of chirality to transform from the sp2 hybridized carbon atom into an sp3 one. With this method, it is possible to reduce the carbonyl group and produce asymmetric hydrogenation and oxidation of alkenes [65].

**FIGURE 21:**
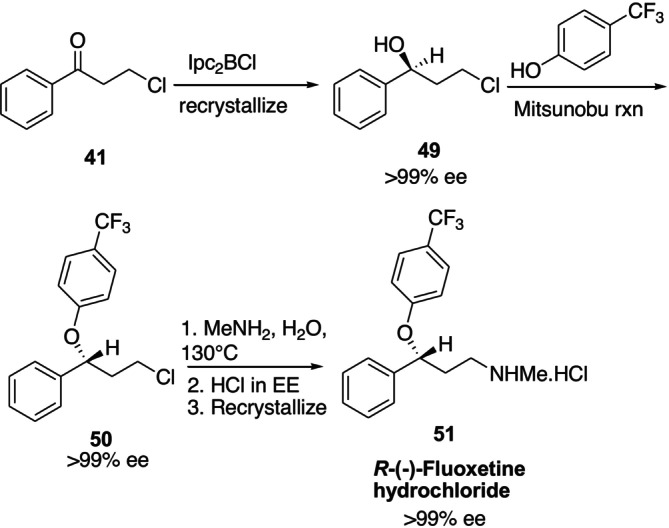
Application of Ipc_2_BCl in the synthesis of *R*‐(−)‐fluoxetine hydrochloride.

Organoboranes are useful compounds that can be utilized as chiral reagents to create a variety of chiral drugs or drug intermediates [66, 67, 68]. In organic synthesis procedures, β‐chlorodiisopinocampheylborane (Ipc_2_BCl) is widely utilized while reducing aralkyl or a‐hindered ketones. In Figure 21, it is shown how this reagent was utilized in the first enantioselective synthesis of the frequently utilized antidepressant (*R*)‐(−)‐fluoxetine hydrochloride **51** [66, 69].

**FIGURE 22:**
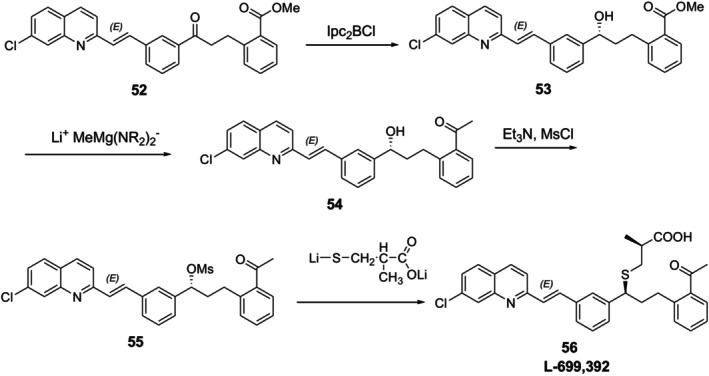
Asymmetric synthesis of a leukotriene antagonist L‐699,392.

In another example, King et al. reported the asymmetric synthesis of L‐699,392 **56**, also known chemically as [3‐[[(l*S*)‐[3(E)‐[2‐(7‐chloroquinolinyl)ethenyl]phenyl]‐3‐(acetylphenyl)propyl]thio]‐2(*S*)‐methylpropanoic acid], a leukotriene antagonist [70]. Using optically active Ipc_2_BCl, the ketone **52** is chirally reduced to create the asymmetric center (Figure 22).

#### 2.2.3 Chiral Catalyst

Asymmetric synthesis utilizing chiral catalysts offers some advantages compared to chiral auxiliaries and reagents. A small amount of chiral catalyst is adequate to produce enantiomerically pure or enhanced products from diverse substrates. Compared to chiral auxiliaries and reagents, chiral catalysts are less expensive. In addition, the modification of the substrate is not necessary for chiral catalysis, unlike chiral auxiliaries. Biocatalysis, metal catalysis, and organocatalysis are the three bases of asymmetric catalysis [71].

##### 2.2.3.1 Biocatalysis

Microorganisms and enzymes (biocatalysts) exhibit excellent enantio‐, chemo‐, and regioselectivity under a wide variety of reaction circumstances. Such high selectivity is frequently sought in pharmaceutical synthesis due to its advantages, including minimizing side effects, preventing the requirement for protection and deprotection steps, and enabling shorter synthesis times. On an industrial scale, biological catalysts, such as isolated enzymes or entire cells, are commonly employed and are especially preferred for hydrolytic reactions. In reality, biocatalysis has been used to successfully synthesis a number of significant medicinal agents, including sitagliptin, pregabalin, zanamivir, montelukast, ragaglitazar, paklitaksel, abakavir, atazanavir, atorvastatin, rosuvastatin, and omapatrilat. The literature provides a summary of the applications of biocatalysis in the synthesis of diverse pharmacological drugs [72, 73, 74, 75]. A few examples are given below.

**FIGURE 23:**
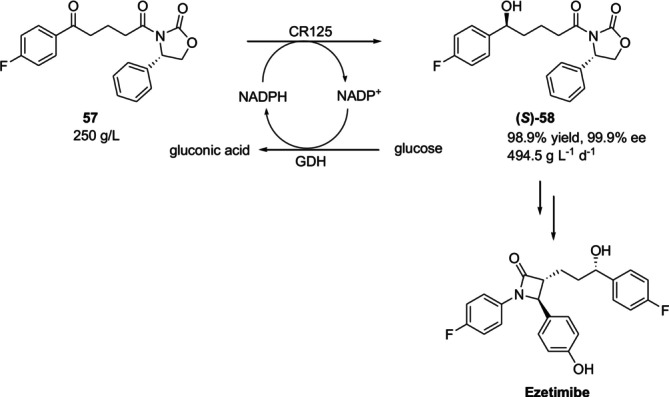
CR125‐catalyzed reduction of 56 to (*S*)‐58, a synthetic intermediate to ezetimibe.

Ketoreductases (KREDs), also known as carbonyl reductases or alcohol dehydrogenases, belong to a class of enzymes that can stereoselectively reduce prochiral ketones to chiral secondary alcohols using reduced nicotinamide adenine dinucleotide phosphate (NADPH) or reduced nicotinamide adenine dinucleotide (NADH) as the hydride donor [76]. Ezetimibe is an antihyperlipidemic drug that selectively prevents cholesterol uptake. Moreover, it is employed in the prevention and management of cardiovascular disorders such as atherosclerosis, coronary heart disease, and others. Zhang et al. recently reduced the ketone precursor **57** to chiral alcohol **(*S*)‐58**, a crucial synthetic intermediary to ezetimibe, through the use of a recombinant *E. coli* strain coexpressing the ketoreductase CR125 from *Lactobacillus kefiri* and GDH for the first time (Figure 23) [77].

**FIGURE 24:**
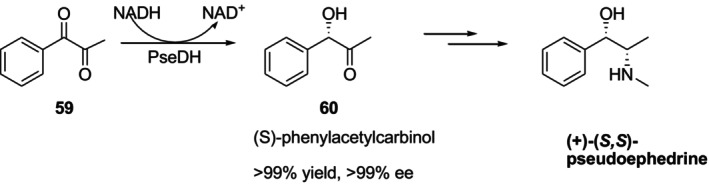
PseDH‐catalyzed reduction of **59**–**60**, a synthetic intermediate to (+)‐(*S,S*)‐pseudoephedrine.

A useful intermediary used in the production of many medicines, including (+)‐(*S,S*)‐pseudoephedrine, is (*S*)‐phenylacetylcarbinol **60**. In 2019 Shanati et al. reported that they reduced 1‐phenyl‐1,2‐propandione **59** to (*S*)‐phenylacetylcarbinol **60** under the catalysis of pseudoephedrine dehydrogenase named PseDH isolated from *Arthrobacter* sp. TS‐15. This reaction produced **60**, the intermediate of (+)‐(*S,S*)‐pseudoephedrine synthesis at > 99% ee to > 99% yield (Figure 24) [78].

**FIGURE 25:**
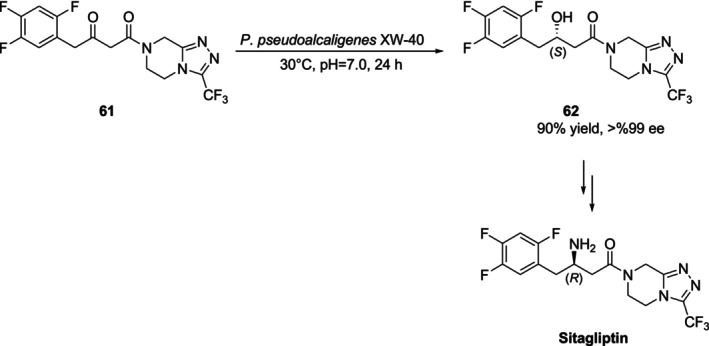
Synthesis of a chiral intermediate 61 of sitagliptin with high enantioselectivity by microbial bioreduction.

In 2016, Wei et al. produced an important intermediate **62** for the synthesis of sitagliptin, a drug used to treat type 2 diabetes [79], using *Pseudomonas pseudoalcaligenes* XW‐40 ( *P. pseudoalcaligenes* XW‐40) [80]. The preparative‐scale microbial bioreduction produced **62** at > 99% ee and 90% yield under ideal circumstances (Figure 25).

Biocatalysis generally provides superior chemo‐, regio‐, and enantioselectivity under mild reaction conditions. However, it may be limited by narrow substrates, enzyme instability, and the requirement for specific cofactors. It might be limited, however, by a narrow range of substrates, enzyme instability, and the need for specific cofactors [81].

##### 2.2.3.2 Metal Catalysis

**FIGURE 26:**
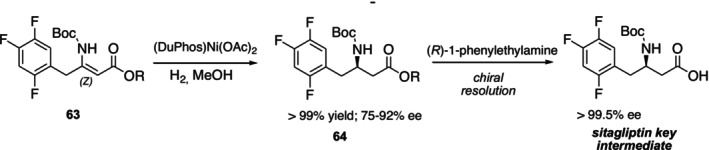
Asymmetric synthesis of sitagliptin intermediate by nickel‐catalyzed asymmetric hydrogenation.

Metal‐catalyzed asymmetric reactions generally offer wide substrate compatibility, high reaction rates, and excellent enantioselectivity. These advantages make metal catalysis suitable for both laboratory and industrial‐scale applications [82]. In asymmetric synthesis, various transition metals such as copper, nickel, iron, and palladium are commonly used as catalysts [83]. Recently, Sudhakaran et al. successfully synthesized the sitagliptin intermediate 3‐*R*‐Boc‐amino‐4‐(2,4,5‐trifluorophenyl)butyric ester **64** via nickel‐catalyzed asymmetric hydrogenation with > 99% yield and 75%–92% enantiomeric excess. While 3‐*S*‐Boc‐amino‐4‐(2,4,5‐trifluorophenyl)butyric acid was effectively resolved using a chiral resolving agent (*S*)‐1‐phenylethylamine to get > 99% ee, 3‐*R*‐Boc‐amino‐4‐(2,4,5‐trifluorophenyl)butyric acid was partially enantioenriched and had 76% ee (Figure 26) [84].

**FIGURE 27:**
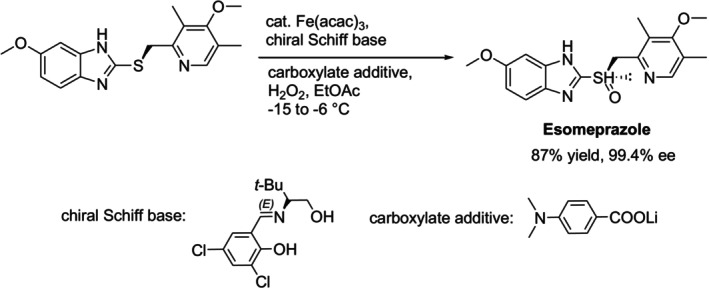
Esomeprazole synthesis catalyzed by the Fe (ııı)‐chiral Schiff base complex.

Iron(I) is employed as a catalyst for the commercial synthesis of drug ingredients because of its low toxicity and easy availability [85]. In 2018, Nishiguchi et al. reported employing an iron salt/chiral Schiff base in conjunction using a carboxylate additive to catalyze the kilogram‐scale asymmetric synthesis of the proton pump inhibitor, esomeprazole, with an 87% yield and 99.4% efficiency (Figure 27) [86].

**FIGURE 28:**
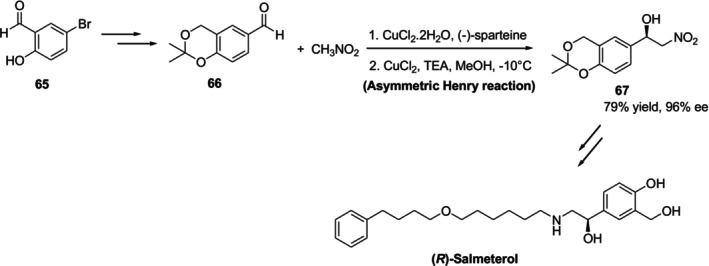
Synthesis of (*R*)‐salmeterol involving a metal‐catalyzed asymmetric Henry reaction.

A technique for the enantioselective synthesis of (*R*)‐salmeterol was described by Guo et al., and a crucial step in it is the Cu (II)‐spartin complex‐catalyzed asymmetric Henry reaction. It was reported that (*R*)‐salmeterol was achieved in 39% overall yield and 95% enantiomeric excess (%ee) in the method (Figure 28) [87].

##### 2.2.3.3 Organocatalysis

**FIGURE 29:**
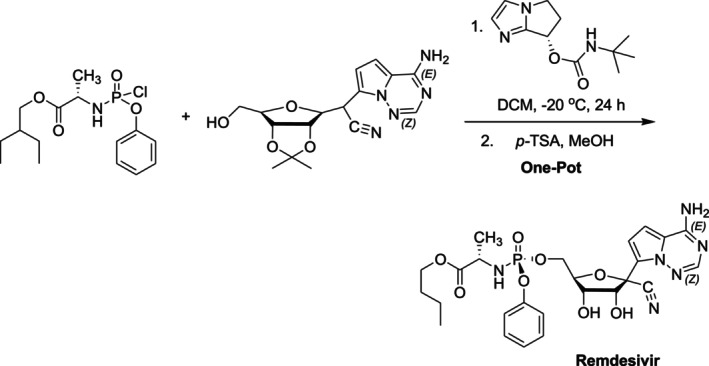
Remdesivir synthesis through one‐pot organocatalyzed asymmetric (*S*)‐P‐phosphoramidation.

Transition metal catalysts are especially useful for asymmetric hydrogenations, but they can also leave dangerous heavy metal traces in the finished product. In this regard, organocatalysts are increasingly being used in place of catalytic metal complexes in chemical science and the pharmaceutical sector. In organocatalysis, a small molecule that is entirely organic and devoid of metals is utilized to catalyze a chemical reaction. In general, organocatalysts are stable substances that are widely accessible on the market or that are simple to synthesize. They are easily connected to a solid support and are frequently based on nontoxic substances like sugars, peptides, or even amino acids. This makes them suitable for industrial applications [71, 88]. Organocatalysis has been successful in the synthesis of several antiviral drugs, such as Oseltamivir (Tamiflu), Zanamivir (Relenza), and Efavirenz (Stocrin). This method has also been used to manufacture numerous additional pharmaceutical substances, such as paroxetine, warfarin, and pregabalin, and comprehensive reviews on the subject have recently been published [89, 90, 91]. In 2025, Jin and colleagues reported the synthesis of enantioenriched compounds with antiproliferative potential via chiral squaramide‐catalyzed enantioselective Friedel–Crafts reactions between phenanthrenequinones and indole derivatives [92]. In 2021, Gannedi et al. developed an organocatalyst with an imidazole‐cinnamaldehyde derivative chiral carbamate structure for the synthesis of the antiviral drug Remdesivir. Using this catalyst, Remdesivir was synthesized in a single vessel with high yield and enantioselectivity by first asymmetric (*S*)‐phosphoramidation followed by acidic hydrolysis to remove the isopropylidene protecting group (Figure 29) [93].

The use of flow technologies, especially in asymmetric synthesis using chiral catalysts, has grown in popularity in recent years. Large amounts of catalyst are usually needed for organocatalysis, and further purifying procedures are necessary for catalyst recovery. In this context, the integration of organocatalysis with continuous flow technologies offers a promising approach for reducing catalyst load and facilitating its recovery [94].

Continuous flow enantioselective catalysis has found a wide range of applications in the asymmetric synthesis of various pharmaceutically active substances. Continuous flow reactors provide for better control of crucial reaction parameters including temperature, residence time, and mixing. They also improve enantioselectivity by considerably improving heat and mass transport. These methods also make it easier to scale and enable safer management of toxic or reactive intermediates [95, 96, 97]. For example, Zhang and colleagues performed an asymmetric synthesis of cyproterone acetate with an overall yield of 9.6% in about 3 h, using 4‐Androsten‐3,17‐dione as the starting material. This was accomplished through a 10‐step chemo‐biocatalytic process that proceeded entirely under continuous flow conditions, eliminating the need for intermediate isolation and purification [98]. Furthermore, there are studies demonstrating that integrating continuous flow with electrochemistry offers a novel way to perform enantioselective transformations in a controlled, secure, and repeatable manner [99]. For instance, in the multistep synthesis of (−)‐oxycodone, Opatz et al. reported achieving high selectivity by electrochemical diastereoselective anodic oxidations conducted under continuous flow conditions [100].

In recent years, there have been interesting studies on the use of ML algorithms as a tool in catalyst selection in asymmetric synthesis. For example, Baczewska and colleagues demonstrated that suitable catalysts can be suggested for magnesium‐catalyzed asymmetric reactions using a ML model based on literature data, and they also reported that the model suggestions were successfully experimentally validated, particularly in synthetically challenging asymmetric reductions and Michael additions [101]. Similarly, Das and colleagues developed a ML model for predicting enantioselectivity in relay Heck (RH) reactions, a significant class of catalytic asymmetric transformations [102]. These studies offer a new perspective for developing more cost‐effective and time‐efficient reaction development strategies in asymmetric catalysis.

In addition to traditional catalytic strategies, electrochemical approaches have recently gained attention in asymmetric synthesis. Electrosynthesis allows redox transformations to occur under mild conditions without the need for strong oxidizing or reducing agents. Asymmetric electrosynthesis is still under development. However, a comprehensive overview of its applications in the synthesis of biologically active compounds is presented in a recent review [103].

Overall, organocatalysis provides a versatile and metal‐free alternative for asymmetric synthesis, offering advantages due to its reliability and effectiveness across diverse substrates. Nevertheless, it generally requires higher catalyst loadings and exhibits lower selectivity compared with biocatalysts [89].

## 3 Racemic Resolution Methods

Racemic resolution continues to be the most popular technique for commercial synthesis of a single enantiomer despite the enormous advances in asymmetric synthesis. Resolution is based on the creation of transitory or covalent diastereomers between a chiral selector and a racemic drug. It is simple to distinguish between the diastereomers since they have diverse physical, chemical, and optical characteristics [104]. While selecting the best chiral resolution technique, there are a few things to take into account. A little amount of a racemic compound is required to assess pharmacological activity early in the drug discovery process. Both enantiomers must be examined for biological testing after the most active molecule has been determined. At this moment, a minimum of 10 mg of the isomer is necessary [105]. In general, this can be accomplished using chromatographic methods like high performance liquid chromatography (HPLC) and supercritical liquid chromatography (SFC). On the other hand, diastereomeric crystallization, kinetic resolution, and simulated moving bed (SMB) are predominantly preferred in kilogram or ton scale productions in the industry. For the preparative scale separation of enantiomers, a number of enantiomeric separation technologies have been developed, including membrane separation, enantioselective liquid–liquid extraction, chiral resolution based on crystallization, kinetic resolution, and HPLC [106]. Commonly used racemic resolution methods are summarized below.

### 3.1 Crystallization Methods

#### 3.1.1 Diastereomeric Crystallization

This technique, which is based on an acid–base reaction between the racemic mixture to be separated and a chiral molecule (resolving agent), is also known as classical resolution or chemical resolution. The racemate to be resolved is transformed into a pair of diastereomeric salts with distinct solubilities by reaction with an appropriate resolving agent and is subsequently separated by crystallization. It is accomplished by adding chiral resolving agents to racemic mixtures, which are often chiral acids or bases. Although diastereomeric crystallization is a straightforward and age‐old technique, it is still frequently used to prepare the vast majority of chiral bioactive compounds needed in enantiopure forms for the production of pharmaceuticals [106, 107]. For instance, thousands of tons of (*S*)‐naproxen are manufactured every year. Table 1 displays a list of a few chosen drugs and the related resolving agents [108, 109].

**TABLE 1:** Drugs prepared via diastereomeric crystallization.

Chiral drugs	Resolving agent	Pharmacological activity
Ethambutol	L‐(+)‐Tartaric acid	Tuberculostatic
Chloramphenicol	D‐Camphosulfonic acid	Antibiotic
Ampicinin	D‐Camphosulfonic acid	Antibiotic
Naproxen	Cinchonidine	Anti‐inflammatory
Diltiazem	*R*‐(+)‐Phenethylamine	Calcium channel blocker
Fosfomycin	*R*‐(+)‐Phenethylamine	Antibiotic
Pregabalin	(*S*)‐Mandelic acid	Antiepileptic
Duloxetine	(*S*)‐Mandelic acid	Antidepressant
Eszopiclone	(+)‐Dibenzoyl‐D‐tartaric acid	Sedative

**FIGURE 30:**
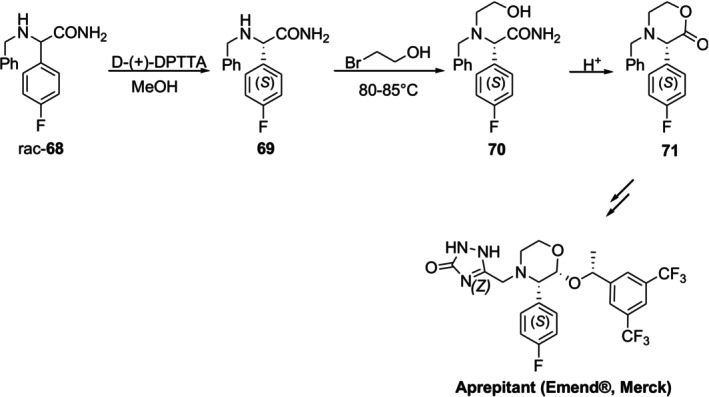
Synthesis of enantiopure (*S*)‐(+)‐71, a crucial intermediate of aprepitant (Emend), through diastereomeric crystallization.

Aprepitant (Emend, Merck), an NK1 receptor antagonist, is employed to treat nausea and vomiting after chemotherapy and surgery. Kolla et al. reported an efficient method based on classical resolution to produce enantiopure (*3S*)‐4‐benzyl‐3‐(4‐fluorophenyl)morpholin‐2‐one **71**, which is a crucial intermediate in the synthesis of aprepitant (Figure 30). In this method, *N*‐benzylglycinamide rac‐**68** was separated by diastereomeric salt crystallization using (+)‐di‐*p*‐toluoyl‐D‐tartaric acid (DPTTA) as the resolving agent and (*S*)‐(+)‐**69** was obtained. The required enantiomer (*S*)‐(+)‐**71** was produced with good yields by alkylating (*S*)‐(+)‐**69** with 2‐bromoethanol and then stereo‐controlled cyclizing the resulting (*S*)‐(+)‐**70** [110].

**FIGURE 31:**
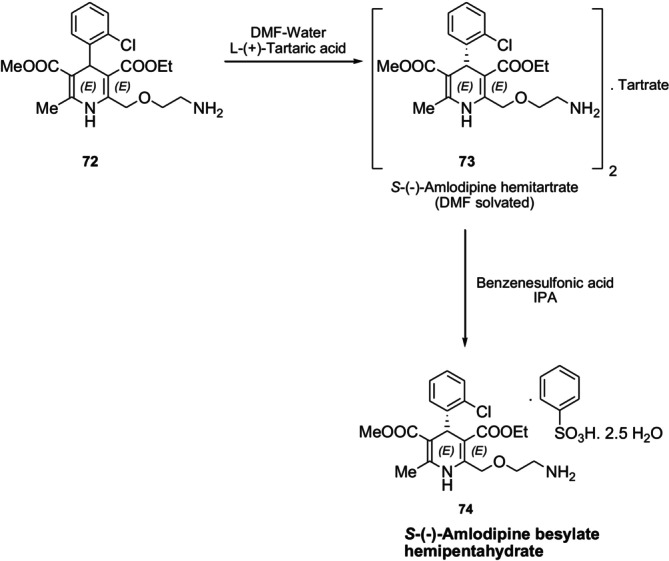
Resolution of racemic amlodipine 72 with L‐(+)‐tartaric acid.

In contrast to the *R*‐isomer, *S*‐amlodipine and its salts are long‐acting calcium channel blockers that can be used to treat cardiovascular diseases. In 2010, Gotrane et al. developed a method using inexpensive, naturally occurring tartaric acid for the synthesis of pure *S*‐amlodipine besylate hemipentahydrate **74**. Amlodipine **72** is directly reacted with natural L‐tartaric acid in DMF to produce *S*‐form amlodipine tartrate **73**, which is the desired product. Consistent *S*‐amlodipine purity (+99%) and acceptable solubility efficiency were obtained by optimizing this method by changing the amount of water in the DMF (Figure 31) [111].

**FIGURE 32:**
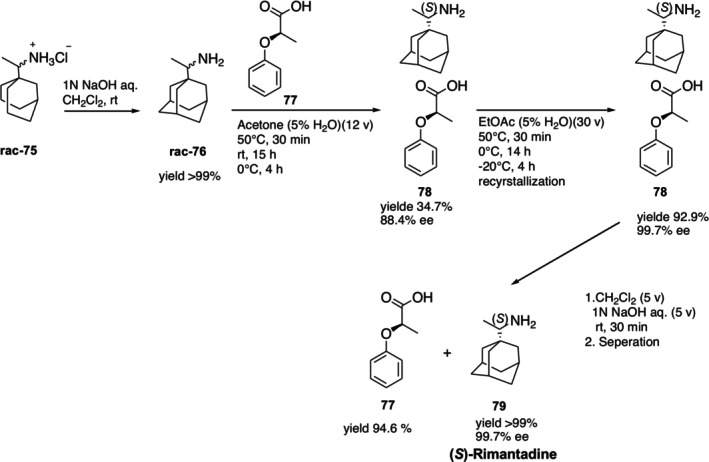
Preparation of (*S*)‐rimantadine from racemic rimantadine hydrochloride using (*R*)‐phenoxypropionic acid as the resolving agent.

In another study by Han et al., (*R*)‐phenoxypropionic acid was used as a reusable resolving reagent to transform rimantadine hydrochloride into pure (*S*)‐rimantadine. In the method, after the commercially available racemic rimantadine hydrochloride **75** was converted to the racemic free amine form, it was treated with (*R*)‐phenoxypropionic acid **77** in aqueous (5%) acetone and diastereomeric salt **78** was obtained in 34.7% yield and 88.4% enantiomeric excess. The diastereomeric salt 78 was then recrystallized twice with aqueous ethyl acetate to give a 92% yield with 99.7% ee. In the final step, salt 78 was treated with NaOH and extracted with dichloromethane. With a virtually quantitative chemical yield and without sacrifice (99.7% ee) enantiomeric purity, this process produced target free (*S*)‐rimantadine **79**. Resolving reagent 77 was also successfully recovered with a high yield [112] (Figure 32).

#### 3.1.2 Preferential Crystallization

Preferential crystallization, also known as resolution by entrainment, is frequently utilized in the industrial manufacturing of chiral pharmaceuticals like α‐methyl‐L‐dopa [113], chloramphenicol [114], milnacipran [115], etodolac [116], and albuterol [117]. Technically, only racemates (conglomerates) containing mechanical mixing of equal proportions of crystals of the two enantiomers are suitable for use with this approach. Unfortunately, only a small (5%–10%) percentage of medication active components are found in this manner [118]. The conglomerate racemic mixture needs to be more soluble than each enantiomer for the approach to work. Unlike diastereomeric crystallization, preferential crystallization uses an enantiopure seed crystal to selectively crystallize a single enantiomer from a supersaturated racemate without the need of chiral excipients. The seeded enantiomer crystallizes while the opposing enantiomer stays in solution phase thanks to the kinetic advantage that the energy required to develop existing crystals is less than the energy required to crystallize the unseeded enantiomer. The crystallization must be terminated at a point suitable to the individual system before the probability of the opposite enantiomer nucleating increases [119, 120].

Seeded isothermal preferential crystallization is the name of the earliest version of the original preferential crystallization technique (SIPC) [119]. In this method, a supersaturated solution is seeded at a constant temperature in order to crystallize the pure enantiomer. Numerous generations of the process of preferential crystallization have been developed, including auto‐seeded polythermic programmed preferential crystallization (AS3PC) [121], second‐order asymmetric transformation (SOAT) [122], and auto‐seeded preferential crystallization induced by solvent evaporation (ASPreCISE) [123]. The mother liquor is recycled during the preferred crystallization process in each of these methods, which yields high enantiomer recovery.

In 2022, Sun et al. reported a study reporting that preferential crystallization of racemic compounds can be promoted by special additives. In the study, the preferential crystallization of norvoline, a key intermediate in the synthesis of perindopril used in the treatment of essential hypertension, was investigated in the absence and presence of special additives. It was found that the purity and yield of L‐norvaline preferential crystallization could be significantly improved up to 98% and 56%, respectively, using the additive‐assisted approach [124].

Although one of the most established methods in industry, diastereomeric crystallization has some significant disadvantages. Using a suitable chiral solvent agent in stoichiometric quantities can increase the difficulty of the synthetic process, leading to additional purification and recovery steps. Furthermore, the theoretical yield is limited to 50%. In contrast, preferential crystallization is cost‐effective because it provides a kinetically controlled separation by the addition of a pure seed crystal of the compound without the use of a chiral resolution agent. Pure enantiomers are obtained directly without the need for additional chemical processes. However, temperature and time require precise control. Furthermore, the applicability of this method depends on the compounds having a “conglomerate” structure, which is rare in nature.

#### 3.1.3 Co‐Crystallization

Although preferential crystallization is an effective method, its applicability to only a small number of pharmaceutical compounds that form conglomerates limits its use. Chiral co‐crystallization offers an alternative chiral separation method for compounds that are not amenable to preferential crystallization and cannot form diastereomeric salts. In this method, usually one of the two enantiomers is selectively co‐crystallized with chiral resolving agents. Chiral resolution via cocrystallization resembles diastereomeric crystallization in that an additional chiral molecule is introduced to generate a novel crystal product with a specific enantiomer; however, the underlying mechanism differs. In diastereomeric crystallization, the chiral compound and the chiral resolving agent interact through strong ionic bonds, while in cocrystallization, the interaction is through hydrogen bonding, van der Waals forces, π−π interactions, and halogen bonds. Thus, the compound is not derivatized and its biological activity is preserved. Furthermore, because of the weaker bonding in cocrystals, their separation is far simpler than diastereomeric salts [125, 126].

**FIGURE 33:**
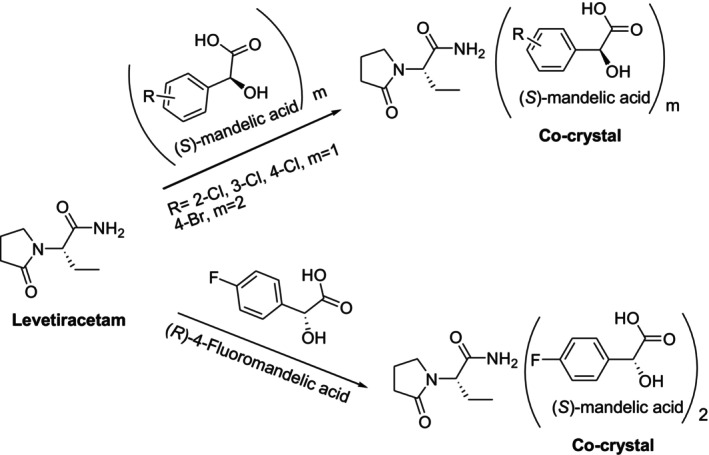
Co‐crystal behavior of levetiracetam and halogenated mandelic acid enantiomers enantioselectively.

Recently, research in the pharmaceutical industry has focused on creating enantioselective co‐crystals. By using (*S*)‐mandelic acid as a co‐crystal former (or co‐former), Springuel et al. were able to resolve (*R,S*)‐2‐(2‐oxopyrrolidin‐1‐yl) butyramide and get antiepileptic drug levetiracetam, namely, (*S*)‐2‐(2‐oxopyrrolidin‐1‐yl) butyramide, in a 70% yield [127]. Wang et al. used halogenated mandelic acids to study the resolution of levetiracetam via cocrystallization in 2021. According to the study, 4‐fluoromandelic acid co‐crystallizes with the *R* enantiomer, whereas levetiracetam co‐crystallizes with the *S* enantiomer of 2‐chloro, 3‐chloro, 4‐chloro, and 4‐bromomandelic acid (Figure 33). Under ideal circumstances, the resolution efficiency with 3‐chloromandelic acid was reported to be as high as 94% [128].

The *S*‐enantiomer of *RS*‐ofloxacin is substantially more potent than the racemic combination, with potencies ranging higher from 8 to 128 times. For the chiral separation of *RS*‐ofloxacin, Kaviani et al. created a novel diastereomeric cocrystal production technique in 2025 using L‐glutamic acid. During this process, cocrystals of *R* and *S*‐ofloxacin were produced by evaporative crystallization, and Fourier transform infrared spectroscopy (FTIR) and powder x‐ray diffraction (PXRD) and melting point analyses revealed structural changes confirming cocrystal formation. Capillary electrophoresis (CE) was used to identify the best solvent mixture for diastereomeric pair separation, and an evaporative crystallization method in methanol: chloroform (50:50, v/v) produced the stronger (*S*)‐enantiomer with a reported ee of 61.82% [129]. In 2022, Songsermsawad and co‐workers developed a new cocrystallization method for the chiral separation of *RS*‐baclofen and were able to obtain *R*‐baclofen with more than 95% enantiomeric purity and 40.8% yield [130]. In 2020, Buol and co‐workers developed an innovative approach combining preferential crystallization and cocrystallization methods to achieve chiral separation of mandelic acid. Nefiracetam, a nootropic drug, was employed as a coformer in the study. By preferentially crystallizing in acetonitrile, mandelic acid—which was found to form a 1:1 cocrystal conglomerate with nefiracetam—was obtained with 98%–99% enantiomeric purity of the R‐mandelic acid enantiomer [131].

### 3.2 Chiral Chromatography

Chiral chromatography has been proven to be a crucial tool in the drug discovery process, serving both analytical needs (such as enantiomer composition determination, quality control of enantiomeric drugs, or stereoselective pharmacokinetic analysis) and preparative needs (such as providing quick access to individual enantiomers for biological testing). There are different approaches for enantiomeric separation based on liquid chromatography: (i) Enantiomers can be separated chromatographically using achiral stationary and mobile phases after conversion to their diastereomeric derivatives. (Indirect method). (ii) The second approach involves the use of chiral mobile phase additives (CMPA). The distinction is based on the formation of transient and reversible diastereomeric molecular linkages in the mobile phase. As a result, enantiomers show different adsorption on an achiral stationary phase. (iii) The third approach uses chiral stationary phases (CSP) for separation of enantiomers and is currently the most preferred method. (Direct method) [132]. All chromatographic separation techniques rely on the generation of transitory diastereomers between enantiomers of a racemate and a chiral selector. There are numerous ways to apply chiral chromatography. The most popular methods for enantiomer separation for analytical purposes are high performance liquid chromatography (HPLC), gas chromatography (GC), supercritical liquid chromatography (SFC), CE, and capillary electrochromatography (CEC). In the preparative separation of chiral pharmaceuticals, HPLC, SFC, and SMB are dominant methods [133]. The most commonly used enantio‐separation methods in drug research are described below with examples from the literature.

#### 3.2.1 High Performance Liquid Chromatography

HPLC has numerous advantages. The selectivity can be altered based on the mobile phase, and a number of chiral stationary phases and chiral selectors are available. It is suitable for the separation of thermolabile and nonvolatile substances. These factors make it a popular choice for analytical and preparative enantiomer separation in both industry and academic research. Enantiomeric separation by HPLC can be performed using CSPs or CMPAs as previously mentioned. CMPA‐based HPLC utilizes lower‐cost achiral columns and does not require precolumn derivatization. Furthermore, a single column can be utilized with multiple additives simultaneously, and CMPAs can be rapidly switched for different analytes, providing methodological flexibility [134]. However, HPLC with CMPAs is more suitable for analytical purposes. On the other hand, the use of CSPs in which the chiral selector is attached to a stationary phase is very widely used in the industry for the preparative resolution of racemic drugs [135]. There are several different types of CSPs available today, including Pirkle‐type, ion exchange (IE)‐type cyclodextrin‐based, cyclofructan‐based, macrocyclic antibiotics‐based, synthetic polymer‐based (polyacrylamides, polymethacrylates), macrocyclic antibiotics‐based, crown ether‐based, polysaccharide‐based and protein‐based CSPs [136]. The literature contains recent comprehensive reviews of CSP species and their uses in enantioseparation [137, 138].

Because of their extensive chiral recognition and large loading capacity, polysaccharide‐based CSPs are the most often utilized among them [139, 140, 141]. These phases are chemically derivatized with phenylcarbamate, benzoate, or similar functional groups to improve chiral selectivity. These CSPs allow enantiomers to exhibit different retention times through multiple noncovalent interactions such as hydrogen bonding, dipole interactions, and hydrophobic effects, and thus to be separated during chromatography [142]. The first polysaccharide‐based chiral stationary phases have been developed in a coated format, specifically prepared by the physical adsorption of amylose and cellulose derivatives onto a silica surface. While these columns offer high enantioselectivity, the risk of dissolution of the selector layer in strong organic solvents significantly limits solvent usage. Unlike conventional coated columns, the new generation of immobilized CSPs, developed by covalently bonding selectors to the silica surface, has a wide solvent tolerance, allowing operation under normal phase, reverse phase, and also HILIC (hydrophilic interaction liquid chromatography) conditions. Furthermore, the new generation of immobilized CSPs provides superior separation efficiency thanks to their small particle sizes, such as 3 μm [143]. In the study by Merino et al. (2020), a Chiralpak IG‐3 column based on immobilized amylose tris(3‐chloro‐5‐methylphenylcarbamate) with a particle size of 3 μm was used; it demonstrated high column efficiency and resolution in polar‐organic and reversed‐phase modes, as well as under HILIC conditions, providing short analysis times in the enantioseparation of β‐blockers [144]. Below are examples of the enantioseparation of some pharmaceutical compounds using chiral HPLC. In 2018, He et al. achieved chiral preparative separation of the nonsteroidal anti‐inflammatory drug ketorolac with an HPLC method on polysaccharide‐based Chiralcel OJ‐H and using methanol/formic acid (100:0.1, v/v) as the mobile phase [145].

In 2015, Zhang et al. developed a preparative HPLC method of Chiralpak AS‐H for the rapid and efficient separation of Gatifloxacin (GFX) enantiomers, a fluoroquinolone group antibiotic. Due to the limited solubility of GFX, a precolumn esterification strategy was used. Within an hour, 79.2 mg of the *S*‐enantiomer and 69.4 mg of the *R*‐enantiomer could be separated with an enantiomeric excess of more than 99% using the mobile phase of HE/EtOH/DEA (80/20/0.1, v/v/v) [146].

Bupropion is an atypical antidepressant and its (*S*)‐isomer is more biologically active than its (*R*)‐isomer. Kozlov et al. recently reported that by optimizing chromatographic conditions such as mobile phase composition, suitable acid/base ratio, flow rate, and temperature in LarichcShell CF6‐RN column containing *R*‐naphthylethylcarbamate cyclofructan 6 under multimodal elution conditions, they were able to provide the baseline enantioseparation of bupropion [147].

Racemic ethyl 3,4‐dihydro‐2*H*‐1,4‐benzoxazine‐2‐carboxylate **rac‐80** is a crucial synthon for the development of potent therapeutic medicines. In 2021, Pham et al. conducted the first enantioselective synthesis of **rac‐80** and used an analytical HPLC approach to evaluate the racemization rate of the reaction product. In parallel, enantioseparation of rac‐80 was carried out by preparative HPLC; (*R*)‐ and (*S*)‐enantiomers were obtained from 12 g of the synthesized racemate in high enantiopurity (ee > 99.5%) by preparative HPLC. A preparative Lux‐Cellulose‐2 CSP (250 × 10 mm, 5 m), hexane/EtOH (70/30, v/v) as the mobile phase, and a flow rate of 5 mL/min are used in this economical method under UV detection at 254 nm. With great purity (ee > 99.5%), the two enantiomers of rac‐80 were isolated and collected in two fractions totaling around 5.7 g each [148].

Solifenacin is an antimuscarinic drug used to treat symptoms of overactive bladder, such as frequent urination and urinary incontinence, and bladder muscle spasms. Solifenacin is accessible as succinate salt and its chemical name is (*R*)‐quinuclidin‐3‐yl (*S*)‐1‐phenyl‐3,4‐dihydroisoquinoline‐2(1H)‐carboxylate succinate salt (1:1). The 3*R*,1*S*‐enantiomer (*S,R*‐isomer) of solifenacin is the therapeutically active form, whereas the other enantiomer form (*R,S*‐isomer) has negative effects. In 2023, Vadagam et al. used normal‐phase HPLC with a CSP containing amylose tris (3,5‐dimethylphenylcarbamate) coated on silica gel (Chiralpak, AD‐H) to successfully separate and simultaneously quantify solifenacin (*S, R‐*enantiomer), *R,S*‐enantiomer impurity, and diastereomer impurities (*S,S‐*isomer and *R,R*‐isomer). Using an isocratic elution mode with a mobile phase made up of “*n*‐hexane, ethanol, and diethylamine,” the separation was completed in 35 min with a detection wavelength of 220 nm [149].

#### 3.2.2 Supercritical Liquid Chromatography

Supercritical fluid chromatography (SFC) is one of the most attractive chromatographic techniques for obtaining quick enantioseparations with great productivity. In the pharmaceutical industry, SFC is widely used in the enantio‐separation of many drugs, including chiral vitamins, nonsteroidal anti‐inflammatory drugs, proton pump inhibitors, and beta‐blockers [150, 151, 152]. In SFC, the mobile phase is supercritical fluids that have intermediate characteristics between gas and liquid. Supercritical fluids as eluents for chromatographic separations were initially proposed by Klesper et al. in 1962 [153]. However, the first chiral separation in SFC was carried out in 1985 by Mourier et al. [154]. A pure substance is transformed into a supercritical fluid when the temperature and pressure are increased to or above its thermodynamic critical point. Compared to the liquid or gas phase, a supercritical fluid has distinct properties. The supercritical fluid has a larger density than the gas state, indicating a higher solvating power. It also has a lower viscosity and a higher diffusivity than the liquid state, indicating that it produces less pressure across a column and migrates more quickly. The most widely used supercritical fluid is carbon dioxide (CO_2_). Because its critical temperature (31°C) and pressure (73 atm) are comparatively low. Analyzing thermo‐labile chemicals is possible because of the low critical temperature. By reducing the pressure, CO_2_ can be easily removed after analysis. This implies a considerable decrease in waste output and disposal. In addition, carbon dioxide is inert and harmless, relatively inexpensive compared to organic solvents [155]. However, under supercritical conditions, carbon dioxide is considered a nonpolar solvent with polarity comparable to that of hydrocarbons such as heptane, so the solubility of polar compounds in SFC is limited. To increase the elution power of the mobile phase, modifiers such as acetonitrile, methanol, and ethanol must be added. Modifier concentration is kept to less than 50%. One reason is that employing a higher concentration of modifier may modify the mixture's critical point and inhibit the mobile phase from demonstrating supercritical fluid characteristics. Another reason is that polar modifiers already have the ability to significantly increase solvent strength at low concentrations [106, 155, 156]. The HPLC stationary phases can also be applied to SFC. Lately, there have also been CSPs created specifically for SFC. Compared to HPLC, SFC offers faster analysis and equilibration times, more productivity and efficiency, and lower solvent disposal costs. Moreover, SFC typically exhibits greater column loadability than HPLC and generates reduced hazardous waste. Conversely, in contrast to HPLC, the alternatives for modifying separation (selectivity) through the mobile phase in SFC are significantly constrained. The lack of polar supercritical fluid precludes the use of SFC in reversed phase mode [133, 157, 158].

Recently, Folprechtová et al. studied the enantioseparation performance of some psychoactive compounds (benzofurans, phenidines, and phenidates) with superficially porous particle vancomycin‐based CSPs in SFC and HPLC [159]. The results exhibited a significant level of enantioselectivity of vancomycin‐based columns in both chromatographic techniques; 88% of the substances analyzed in SFC and 69% of those evaluated in HPLC were enantioseparated. In 2017, Segawa et al. reported successful enantioseparation of methamphetamine (central nervous system stimulant) in SFC using cellulose‐based CSP. Optimized chromatographic conditions include the use of Trefoil CEL2 column, MeOH with 0.11% NH_3_ as co‐solvent, 1 mL/min flow rate, 10°C column temperature, and 17.2 MPa back pressure [160].

In 2021, Pandya et al. used SFC to investigate the enantioseparation of three β‐blockers, including atenolol, metoprolol, and propranolol, on amylose tris(3‐chloro‐5‐methylphenylcarbamate) immobilized chiral stationary phase. A mixture of CO_2_ and 0.1% isopropyl amine in isopropanol: methanol (50:50, V/V), in a ratio of 75:25 (V/V), was used to obtain the best chromatographic separation. The ideal parameters were 10 L injection volume, 4.0 mL/min constant flow rate, 40°C column temperature, and 220‐nm wavelength. In addition, the system's backpressure was 100 bars and the sample cooler was maintained at a temperature of 10°C. The resolution factors (Rs) and separation factors (a) were found to be higher than 3.0 and 1.5, respectively, at the optimum conditions [161].

**FIGURE 34:**
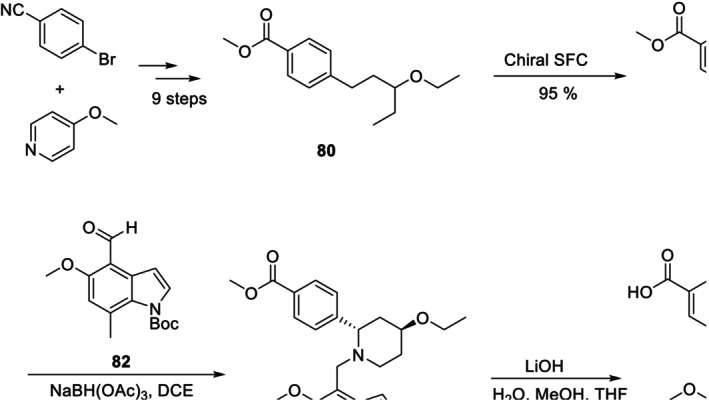
Preparation of Iptacopan.

Iptacopan (Fabhalta, Merck), an inhibitor of complement factor B, is used to treat PNH, or paroxysmal nocturnal hemoglobinia. Iptacopan is synthesized in 12 steps starting from 4‐bromobenzonitrile and 4‐methoxypyridine. The chiral SFC of the intermediate **80** to form **81** is the key step in the asymmetric synthesis of Iptacopan. Iptacopan was obtained via condensation of 81 with 82, followed by ester hydrolysis and N‐deprotection in an alkaline environment (38% yield, 99% ee) (Figure 34) [162].

Recent investigations have explored more environmentally sustainable alternatives to organic modifiers like methanol and acetonitrile in chiral separations utilizing SFC, aimed at enhancing solubility and selectivity. In a study conducted by Jurin et al. in 2024, the enantioseparation of (±)‐trans‐β‐lactam ureas on immobilized polysaccharide‐based chiral stationary phases was evaluated under both HPLC and SFC conditions. In this study, dimethyl carbonate (DMC) was used for the first time in the literature as an alternative organic modifier in SFC mode; it was determined that DMC effectively separated (±)‐trans‐β‐lactam ureas due to its characteristic polarity and hydrogen bonding properties [163].

#### 3.2.3 Simulating Moving Bed Chromatography

SMB, a continuous chromatographic process simulating the countercurrent motion of stationary and mobile phases, was developed by the petrochemical industry in the 1960s and has been widely used in petrochemical and sugar manufacturing processes ever since. Today, it is one of the most important separation methods that allows the separation of racemic pharmaceuticals on an analytical, preparative and industrial scale [164]. It comprises two inlet and exit ports and many connected chromatographic columns. The fundamental distinction between HPLC and SMB chromatography lies in the dynamic flow direction of the desorbent in SMB chromatography, which mimics a countercurrent interaction between the fluid and solid phases. SMB separations outperform single column preparative chromatography in terms of productivity and purity while using less solvent [165, 166].

Levetiracetam (antiepileptic drug) is manufactured by UCB Pharma in Belgium using the SMB process as a single enantiomer. Tens tons of enantiomers with 98.5% purity and satisfactory recovery (90%) are generated annually in this industrial‐scale separation system, which consists of six columns holding a combined total of 50 kg of CSP [167]. In a six‐column laboratory‐scale unit (FlexSMB‐LSRE) packed with Chiralpak AD stationary phase (20 μm), Ribeiro et al. separated flurbiprofen enantiomers preparatively using SMB technology. As a solvent, a mixture of 10% ethanol, 90% *n*‐hexane, and 0.01% TFA was utilized. The purities were reported to be over 99.4% for both output streams [168]. Laurus Labs uses an SMB‐based process to separate S‐efavirenz, which has an enantiomeric purity of over 99.9% and a production capacity of roughly 300 kg per day [169]. In 2016, Chen et al. separated metalaxyl racemate on EnantioPak OD columns using a combination of hexane and ethanol (70:30, v/v), and the purity of each enantiomer product was over 99% [170].

As summarized in Table 2, HPLC and SFC are preferred in the early stages of pharmaceutical drug discovery (library search and lead compound optimization) due to their high resolution, wide range of chiral column options, and method development flexibility. SFC, in particular, offers a sustainable alternative for high‐speed enantiomer screening thanks to its low‐viscosity mobile phase and reduced organic solvent consumption. However, process economics becomes crucial in the transition of the candidate molecule from pilot scale to commercial production; solvent recovery, energy efficiency, and operational continuity stand out as critical parameters. In this context, SMB systems are considered one of the most advantageous approaches on an industrial scale.

**TABLE 2:** Comparison of chromatographic techniques for enantioseparation of pharmaceutical compounds.

Parameter	HPLC	SFC	SMB
**Mobile phase**	Organic solvents (normal‐phase or reversed‐phase systems)	Supercritical CO_2_ + organic modifier (MeOH, EtOH, etc.)	Based on HPLC or SFC mobile phases
**Operating mode**	Batch	Batch	Continuous
**Analytical throughput**	Moderate	High	Not intended for analytical screening
**Solvent consumption**	High	Low	Moderate to low (High recyclability)
**Method development complexity**	Relatively simple	Moderate	Complex process optimization required
**Preparative capability**	Moderate‐high	High	Very high
**Scalability**	Limited by batch operation	Good	Excellent (industrial‐scale production)
**Process economics (large scale)**	Moderate	Good	Highly favorable
**Typical pharmaceutical role**	Early‐stage screening and quality control (QC) analysis	Rapid enantiomer screening and preparative purification	Pilot‐to‐commercial‐scale active pharmaceutical ingredient (API) manufacturing

*Note:* The table was compiled on a critical evaluation of the literature on chiral HPLC, SFC, and SMB technologies in pharmaceutical enantioseparations (Refs.) [17, 138, 171, 172].

### 3.3 Kinetic Resolution and Dynamic Kinetic Resolution

**FIGURE 35:**
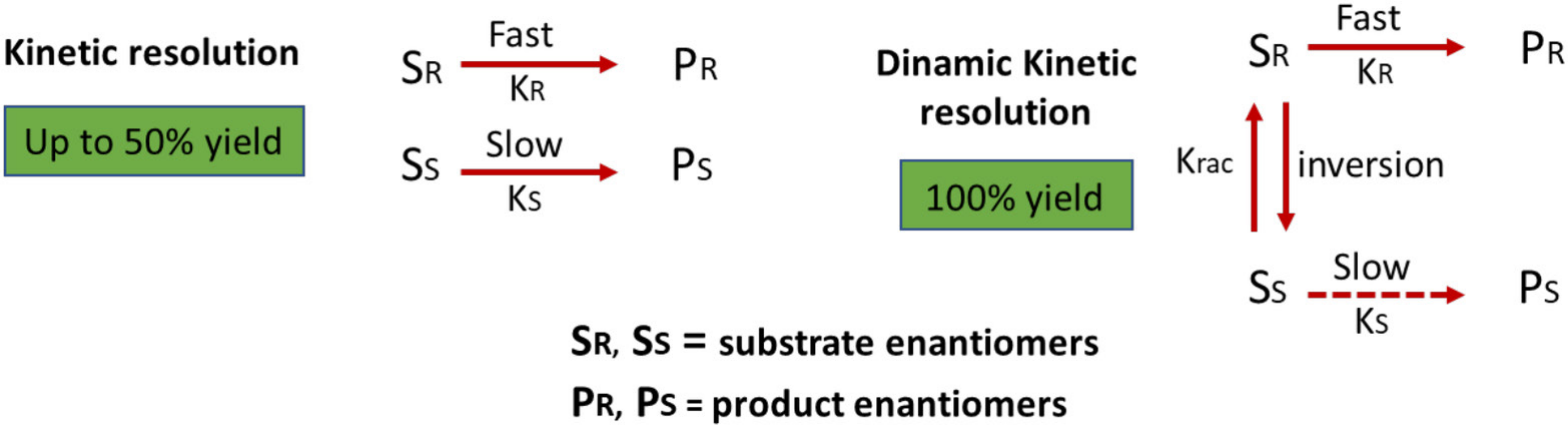
Kinetic and dynamic kinetic resolution.

Kinetic resolution is a well‐established technique that has been used successfully in the synthesis of various organic chiral molecules. The fundamental principle of kinetic resolution lies in the variation between the reaction rates of the two enantiomers with the chiral agent, which may be a biological catalyst (such as enzymes or microorganisms) or a chemical catalyst (such as an acid, base, or metal complex) [173]. An abundance of the less‐reactive enantiomer is produced owing to the difference in reactivity. Choosing the appropriate chiral catalyst for the kinetic reaction is crucial for the success of the method. When KR ≠ KS, kinetic resolution happens and the reaction terminates with anywhere between 0% and 100% conversion (Figure 35). The perfect state is attained when only one enantiomer interacts (KS = 0). In this case, a mixture of *S*‐substrate and *R*‐product is obtained with 50:50. The limitations of the kinetic resolution approach include the requirement to separate the product from the reactants and a maximum conversion in the process of only 50%. The use of dynamic kinetic resolution (DKR) can get around these limitations. DKR facilitates the complete conversion of the achiral reactant, as both enantiomers are interconnected in a chemical equilibrium within DKR. In the course of the resolution procedure, the substrate is constantly isomerized. If the rate of stereoisomerization is sufficiently higher than the rate of conversion to the product, the yield of pure enantiomeric products from the original racemates can theoretically reach 100%.

**FIGURE 36:**
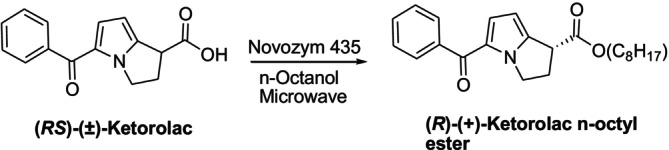
Ketorolac's kinetic resolution by Novozym 435 with microwave assistance.

The literature contains numerous reports of effective enantioseparations of nonsteroidal anti‐inflammatory drugs (NSAIDs) based on kinetic solubility and dynamic kinetic solubility techniques [174, 175, 176, 177, 178, 179]. Microwave irradiation is used in kinetic resolution processes because it is a green application that significantly reduces the time and increases selectivity in biocatalytic conversions [180, 181]. In 2015, Shinde et al. investigated the kinetic solubility of *RS*‐(±)‐ketorolac by different immobilized lipases, including Novozym 435 under microwave heating. Novozym 435 successfully catalyzed the *RS*‐(±)‐ketorolac enantioselective esterification under microwave synergism, achieving good conversion (50%) and enantiomeric excess (> 99%) in 3 h at 50°C and 300 rpm (Figure 36) [179].

**FIGURE 37:**
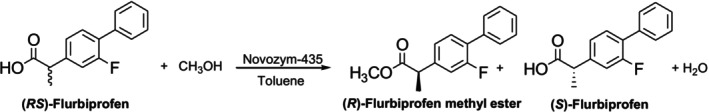
Flurbiprofen's kinetic resolution with microwave aid.

In another study by Gupta et al., the kinetic resolution of (*R,S*)‐flurbiprofen under microwave irradiation using immobilized lipases was investigated. For esterifying the *R*‐enantiomer in toluene, Novozyme 435 has been shown to be the most stable and active biocatalyst. It was shown that microwave radiation and enzyme catalysis work in harmony (Figure 37) [178].

Shiina et al. developed an effective method for nonenzymatic DKR of racemic 2‐arylakanoic acids. The current DKR uses pivalic anhydride, diisopropylethylamine, and benzotetramisole in a polar solvent to enantioselectively esterify racemic 2‐arylalkanoic acids and quickly racemize the chiral 2‐arylalkanoic acids under proper reaction circumstances. The *S*‐enantiomers of several chiral NSAIDs (ibuprofen, naproxen, etc.) were effectively prepared using this approach [176].

**FIGURE 38:**
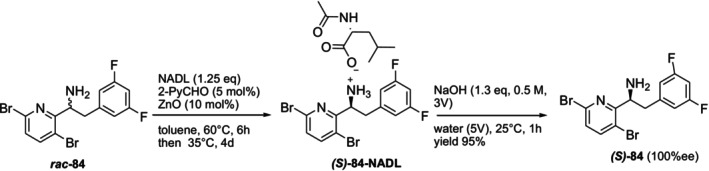
Preparation of enantiomerically pure (*S*)‐1‐(3,6‐dibromopyridin‐2‐yl)‐2‐(3,5‐difluorophenyl)ethan‐1‐amine (S‐84), an important intermediate of lenacapavir by DKR.

In 2024, Shinde et al. developed the DKR approach to obtain the enantiomerically pure (*S*)‐1‐(3,6‐dibromopyridin‐2‐yl)‐2‐(3,5‐difluorophenyl)ethan‐1‐amine (***S*‐84**) compound, an important intermediate of the lenacapavir (anti‐HIV drug). In the resolution process carried out with *N*‐acetyl‐D‐leucine (NADL), (*S*)‐4‐NADL salt was isolated with 61% yield and 99.6% diastereoselectivity (de) in the presence of pyridine‐2‐carboxaldehyde and ZnO catalysts. In the scale‐up study, (*S*)‐4‐NADL salt was obtained with 63% corrected yield and 100% diastereomeric purity using 10 g racemate. In the last stage, the salt was removed with aqueous NaOH and the free (*S*)‐amine was isolated with 95% yield and 100% enantiomeric purity (ee) [182] (Figure 38).

The applicability of kinetic resolution and DKR is determined by the kinetic control of the reaction and the process design requirements. The 50% yield limit of kinetic resolution can mean raw material loss, especially in large‐scale synthesis. However, it is more predictable and efficient because it does not need an additional racemization step. DKR, on the other hand, may theoretically give a full conversion. Nevertheless, for the method to be efficient, the racemization rate has to be far higher than the enantioselective reaction rate. If this kinetic equilibrium is not reached, selectivity may drop, resulting in a mix of enantiomers instead of the chosen enantiomer being formed more often. The use of kinetic resolution and DKR depends on how well the reaction can be controlled and what the process design needs are. The 50% yield limit of kinetic resolution can lead to the loss of raw materials, particularly in large‐scale synthesis. Additionally, in DKR systems that combine metal‐catalyzed and biocatalytic steps, catalyst compatibility is a crucial factor. The risk of mutual deactivation can limit process stability [183].

### 3.4 Enantioselective Liquid–Liquid Extraction

**FIGURE 39:**
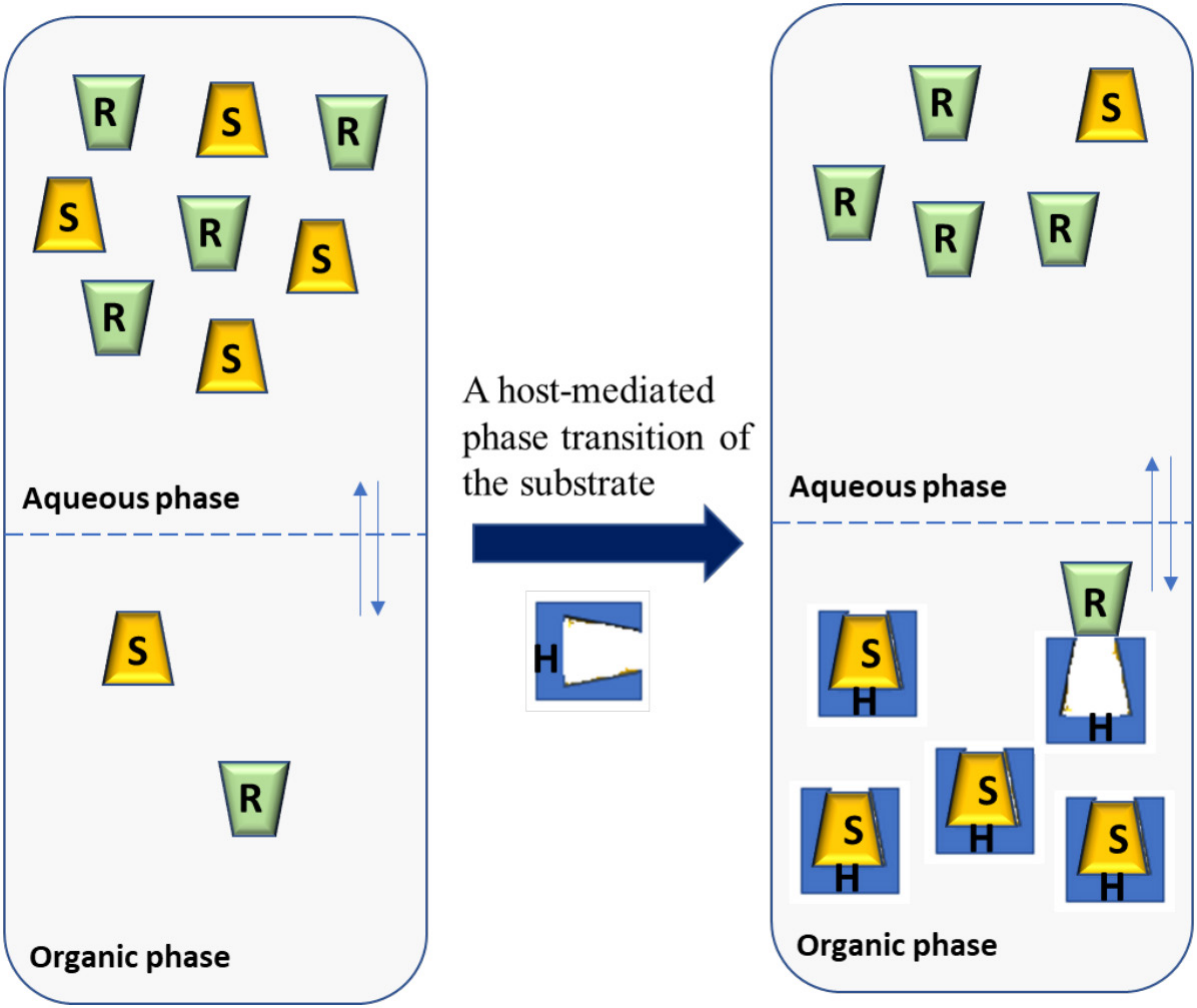
Host‐mediated phase transfer, which is the basic principle of ELLE.

Racemates can be continually separated into their enantiomers using liquid–liquid extraction in a countercurrent manner. The use of liquid–liquid extraction for enantioseparation is appealing due to the flexibility to operate on various reaction scales. Enantioselective liquid–liquid extraction (ELLE) is a technique that combines the ideas of solvent extraction with enantiomeric recognition. ELLE's chiral recognition principle follows the “three‐point interaction” rule. The creation of encounter complexes between the extractant and the enantiomers is caused by weak intermolecular interactions like hydrogen bonding, π–π stacking, dipole–dipole, and van der Waals interactions. A typical ELLE system consists of two immiscible (at least partially) phases, the aqueous and the organic phase, and initially, the substrate is generally kept in the aqueous phase. A host‐mediated phase transition of the substrate takes place after the addition of a lipophilic host or extractant, which prefers to remain constrained in the organic phase [184]. In the example shown in Figure 39, the host interacts more favorably with the (*S*)‐enantiomer than the (*R*)‐enantiomer. As a result, the (*S*)‐enantiomer will be enriched in the organic phase whereas the (*R*)‐enantiomer is enriched in the aqueous phase. The chiral extractant is the crucial component in the ELLE process. Cyclodextrins [185, 186], tartaric acid derivatives [187, 188], crown ethers [189], cinchona alkaloids [190], and metal complexes [191] are currently some of the most common chiral extractants.

In 2013, Peng et al. examined the enantioselective liquid–liquid extraction of Zopiclone, a chiral hypnotic agent belonging to the cyclopyrrolone class, utilizing mandelic acid ester derivatives as extractants. [192]. With a maximal enantioselectivity of 1.6, (*R*)‐o‐chloromandelic acid propyl ester was shown to be an effective chiral extractant for zopiclone resolution. The ideal extraction circumstances were found to be cyclohexane as the solvent, extractant concentration of 0.2 mol/L, ZPC concentration of 0.25 mmol/L, pH of 6.5, and temperature of 5°C.

In 2014, Ren et al. investigated enantioselective liquid–liquid extraction of racemic ibuprofen enantiomers utilizing chiral extractants derived from l‐tartaric acid. The maximal enantioselectivity of racemic ibuprofen enantiomers was greater than 1.2 under the ideal circumstances (0.2 mol of l‐tartaric acid dipentyl ester in the organic phase and decanol as the solvent) [187].

In 2025, Moradi and colleagues developed a liquid–liquid extraction system based on hydrophobic eutectic solvents (HES) to provide the enantioseparation of *RS*‐ofloxacin. In the study, the most effective HES‐based ELLE system was determined to be decanoic acid: dodecanoic acid (C10 acid: C12 acid) in a 2:1 M ratio, and with the optimization of the chiral selector excess, pH and HES‐water ratio (v/v) parameters, they were able to efficiently separate the enantiomers of ofloxacin with a selectivity of 3.8 [193].

Separation efficiency in ELLE depends on distribution coefficients, selectivity factors, and interfacial mass transfer rates. Numerous factors, including solvent polarity, chiral selector structure and concentration, phase ratio, pH, and temperature, influence enantiomer distribution and the formation of chiral host‐guest complexes. Moderate selectivity might require multistage extraction, hence increasing solvent consumption and process complexity. ELLE also has big problems with extractant stability, recyclability, and use on a large scale. Using ML to add to the chiral extractant library is an appropriate approach to improve selectivity and help create new ELLE systems [194].

### 3.5 Membrane Technologies

Membrane‐based enantioseparation techniques have garnered significant attention for industrial applications due to their scalability, minimal energy requirements, capability for continuous operation, and efficiency. Enantioselective membranes serve as selective barriers capable of separating optical isomers because of their chiral recognition sites. There are two types of enantioselective membranes: liquid membranes and solid membranes [195]. A liquid phase that functions as a membrane between two fluid phases makes up liquid membranes. Chiral liquid membrane technology includes the combination of membrane separation and chiral extraction technology. It enables effective concurrent substance extraction and recovery in a single unit process. Racemate separation is achieved by incorporating chiral selectors into the liquid membrane, resulting in the preferential transit of one enantiomer to the membrane holding the selective agent. Solid membranes can be classified as molecularly imprinted membranes, chiral polymer membranes, and achiral support membranes with chiral selectors. The membrane's chiral recognition site interacts with enantiomers to achieve separation. Liquid membranes demonstrate remarkable chiral resolution selectivity and permeability; however, they lack mechanical stability and durability. In comparison to liquid membranes, solid membranes are more suitable for industrial applications owing to their mechanical stability and durability. [196]. There are extensive reviews in the literature on membrane‐based enantioseparations [196, 197, 198, 199, 200, 201, 202].

In the current literature, composite membranes, especially those with chiral covalent organic structures (CCOFs) and metal–organic structures (CMOFs), are becoming more popular. These structures advance beyond just surface interactions by providing intrinsic chiral nano‐channels that make it easier for molecules to match sizes and selectively gate enantiomers [203, 204, 205].

In 2025, Gao and Ben investigated the chiral drug separation performance of CCOF‐300 membranes developed using tartaric acid as a chiral inducing agent. The membrane demonstrated excellent success in separating a large molecule, Fmoc‐Lys (Dde)‐OH, achieving 100% purity (ee) for the first 4 h. However, in experiments with a smaller molecule, Ibuprofen, selectivity remained at 88% in the first hour, and both enantiomers were observed to pass through the membrane. These results clearly demonstrate that the success of chiral separation depends on the size matching between the drug molecule and the membrane pore size. The study offers a low‐cost and effective method, particularly for the purification of large‐structured drugs [203].

## 4 Conclusions

Chirality is very important in drug development because it can change the pharmacodynamic and pharmacokinetic properties in significant ways. The biological activities of chiral drugs' enantiomers can be very different, ranging from beneficial to dangerous. Regulatory bodies like the FDA and EMA are putting more and more pressure on drug companies to develop and approve single enantiomer drugs instead of racemic mixtures. The benefits of single enantiomer drugs, such as better therapeutic effects and fewer side effects and toxicity, support the choice of these drugs.

Asymmetric synthesis and racemic resolution are the two most common methods for obtaining enantiopure drugs. There are many improved asymmetric synthesis methods that are used effectively now in the synthesis of enantioselective compounds. One of which is the chiral pool method; although it provides a sustainable and cost‐effective way to synthesize enantiomerically pure compounds using chiral precursors found in nature, it is limited by the availability of suitable enantiomeric starting materials. On the other hand, chiral auxiliaries provide high stereocontrol; however, they additionally rise in expenses while lowering the yield because they must be used in a certain way and require extra steps to purify. Chiral catalysts (biocatalysis, metal catalysis, and organocatalysis) are better than other types of catalysts because they are more selective and work with smaller amounts of material, which is good for both the economy and the process. In recent years, the integration of continuous flow technologies with ML has shown promise in enhancing efficiency through optimized catalyst selection and reaction conditions. Nevertheless, because of limited datasets and generalizability challenges, ML should be considered a complementary tool for guiding experimental design rather than a method that fully replaces experimental screening.

Racemic resolution, particularly through crystallization‐based techniques, remains a preferred method for large‐scale production of enantiomerically pure drugs. Conventional diastereomeric crystallization is simple and efficient but is only suitable for compounds capable of forming diastereomeric salts. More advanced methods, such as preferential crystallization and chiral co‐crystallization, provide options for compounds that cannot form diastereomeric salts. Preferential crystallization employs seed crystals to obtain pure enantiomers from supersaturated racemates; this approach offers kinetic control but is limited by conglomerate dependency and the need for precise temperature and timing control. Co‐crystallization, in contrast, can be applied to a broader range of compounds without derivatization while preserving biological activity. Modern deracemization strategies, through the simultaneous management of racemization in solution and crystal growth, enable the theoretical production of a single enantiomer from racemic mixtures with 100% yield. DKR serves as a promising alternative for kinetic resolution, continuously converting undesired enantiomers to the preferred form, potentially achieving 100% yield. Emerging methods such as chiral membranes and ELLE attract interest due to their scalability, energy efficiency, and potential for continuous processing, although their performance is highly dependent on solvent type, temperature, pH, and chiral selector concentration, requiring systematic optimization.

Chiral chromatography remains one of the most widely used techniques for both analytical and preparative purposes. HPLC and SFC are valuable tools, offering high resolution and methodological flexibility for early‐stage drug discovery and rapid enantiomer screening, whereas SMB systems provide solvent savings, continuous processing advantages, and cost efficiency at large‐scale production. However, the effectiveness of HPLC and SFC may be limited as scale increases, while SMB faces challenges such as high initial investment and system complexity. Therefore, the selection of chromatographic techniques should be optimized considering production scale, process efficiency, and economic factors.

In the future, ML‐assisted catalyst design and continuous flow technologies are expected to enhance the predictive power of chiral synthesis and resolution strategies, rendering drug development processes more sustainable, efficient, and safe. Research should focus on improving the efficiency of asymmetric catalytic systems, expanding the applicability of chiral resolution techniques, and integrating green chemistry principles into enantioselective processes. Furthermore, a deeper understanding of chiral recognition mechanisms will facilitate the development of novel chiral selectors and catalysts, advancing the field of stereoselective drug synthesis. The pharmaceutical industry's shift towards single‐enantiomer drugs is strongly supported by advances in asymmetric synthesis and racemic resolution. The need for efficient, scalable, and environmentally friendly chiral synthesis and resolution methods will continue to drive innovation in this critical area of drug development.

## Data Availability

The data that support the findings of this study are available on request from the corresponding author. The data are not publicly available due to privacy or ethical restrictions.
